# Anti-Bacterial and Anti-Fungal Activity of Xanthones Obtained via Semi-Synthetic Modification of α-Mangostin from *Garcinia mangostana*

**DOI:** 10.3390/molecules22020275

**Published:** 2017-02-12

**Authors:** Srinivasan Narasimhan, Shanmugam Maheshwaran, Imad A. Abu-Yousef, Amin F. Majdalawieh, Janarthanam Rethavathi, Prince Edwin Das, Palmiro Poltronieri

**Affiliations:** 1 Asthagiri Herbal Research Foundation, 162A, Perungudi Industrial Estate, Perungudi, Chennai 600096, India; asthagiri.herbal@gmail.com (S.N.); mahesh7605@yahoo.co.in (S.M.); rethavathij@gmail.com (J.R.); prince.ahrf@gmail.com (P.E.D.); 2 Department of Biology, Chemistry and Environmental Sciences, American University of Sharjah, P.O. Box 26666 Sharjah, United Arab Emirates; iabuyousef@aus.edu (I.A.A.-Y.); amajdalawieh@aus.edu (A.F.M.); 3 Institute of Sciences of Food Productions, CNR-ISPA, Lecce 73100, Italy

**Keywords:** α-mangostin, anti-bacterial, anti-fungal, packaging, textiles, biomedical device, semi-synthetic modification

## Abstract

The microbial contamination in food packaging has been a major concern that has paved the way to search for novel, natural anti-microbial agents, such as modified α-mangostin. In the present study, twelve synthetic analogs were obtained through semi-synthetic modification of α-mangostin by Ritter reaction, reduction by palladium-carbon (Pd-C), alkylation, and acetylation. The evaluation of the anti-microbial potential of the synthetic analogs showed higher bactericidal activity than the parent molecule. The anti-microbial studies proved that **I E** showed high anti-bacterial activity whereas **I I** showed the highest anti-fungal activity. Due to their microbicidal potential, modified α-mangostin derivatives could be utilized as active anti-microbial agents in materials for the biomedical and food industry.

## 1. Introduction

**Figure 1:**
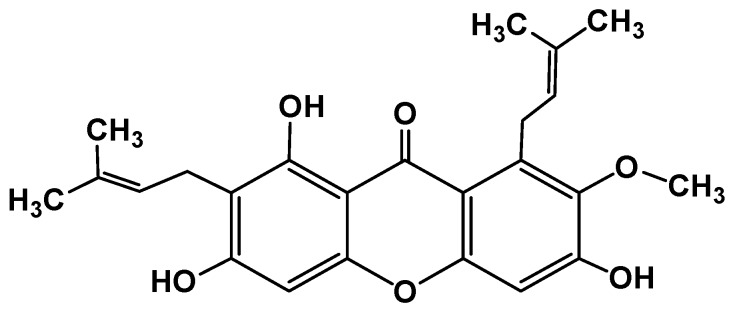
Structure of α-mangostin.

The fruit of *Garcinia mangostana* Linn. (mangosteen), of the family Guttiferae, has been used in Asian traditional medicines for the treatment of skin infections, wounds, diarrhea, dysentery, suppuration, leucorrhea, chronic ulcers, and gonorrhea [1,2]. In addition, mangosteen with essential minerals is commercially used as dietary supplement for cancer patients [3]. The pericarp of the fruit contains high amounts of xanthones, such as α-mangostin (Figure 1), β-mangostin, γ-mangostin, etc., and considerable amounts of other bioactive compounds, such as terpenes, anthocyanins, tannins, flavonoids and polyphenols [4].

Xanthones are naturally-occurring compounds with a distinct chemical structure, known as tricyclic aromatic system, with known antibacterial properties [5]. Natural compounds with antibacterial properties may be applied to treat local infections [5,6,7], wounds and lesions difficult to heal, circumventing antibiotic resistant pathogens with multidrug resistance (MDR) genes, or may be combined with antibiotics to increase their effect. Therefore, studies on bacteria inhibition in vitro or in vivo have been performed on a wide array of natural compounds and peptides [8]. In microbiology, the minimum inhibitory concentration (MIC) is the lowest concentration of a chemical that prevents visible growth of a bacterium (bacteriostatic activity), whereas the minimum bactericidal concentration (MBC) is the concentration that results in microbial death. Among the pathogens developing antibiotic resistances, *Pseudomonas aeruginosa* and *Staphylococcus aureus* (S. *aureus*) are largely spread in hospitals and healthcare units [9,10].

In the literature, mangosteen fruit extracts were shown to contain different xanthones, identified by HPLC analysis: α-mangostin, β-mangostin, γ-mangostin, 8-desoxygartanin and gartanin, two isoprenylated xanthones, and 9-hydroxycalabaxanthone [11]. α-Mangostin (3,6,8-trihydroxy-2-methoxy-1,7-bis(3-methyl but-2-enyl)xanthen-9-one) is a compound purified as a yellow crystalline solid, with molecular mass 410.45 g/mol, having a xanthone core structure. It is prepared by heating of phenyl salicylate (salol). It is used in preparation of xanthydrol, which is used for the determination of urea levels in the blood.

Recent reports have revealed that α-mangostin from *G. mangostana* fruit possesses several medicinal properties, such as anti-microbial activity against bacteria [4,12,13,14,15,16,17,18], anti-oxidant and neuro-protective activity [19,20,21,22], lipase inhibition [23], and anti-inflammatory and anti-cancer properties [24]. Biologically active molecules from medicinal plants are utilized as therapeutic agents, but most of the secondary metabolites do not exhibit optimum efficacy. This is due to the lack of specificity and the absence of biologically active functional groups. Thus, by elucidating the structure of the active compound and the pharmacophores, the functional groups are considered as essential for the bioactivity of a compound. Since α- and non-α-mangostin xanthones have been shown to possess anti-bacterial activity, especially against Gram-positive bacteria [12,13,14,15,16,17,18], it is most probable that semi-synthetic analogs would be produced with enhanced anti-bacterial activity. In order to increase the bioactivity and antibacterial properties of α-mangostin, semi-synthetic modification of the compound was performed by several authors, to lead to more active compounds [25,26,27,28,29], with no excessive toxicity. Alongside their anti-microbial properties, the synthetic analogs possess wound healing and anti-inflammatory activity and, hence, they could be exploited in the treatment of skin infections.

Following the discovery of the medicinal properties of the synthetic analogs, it is suggested that these analogs possess better therapeutic value than the parent molecule and are potential drug candidates for application as antimicrobials. It has been envisaged that mangosteen xanthones may be applied in several fields and industries, such as textiles, fabrics, and polymers for medical devices and biomaterials for applications in biomedicine [30,31,32], in biomaterials for oral hygiene and prevention of dental caries [30], in materials preventing biofilm formation [31], and wrapping foil polymers for food packaging [32]. However, it should be demonstrated that these new compounds are non-toxic and safe, and the derived materials do not release them too fast, possibly being covalently linked, to maintain the bioactivity for longer periods. In addition, pathogens are able to survive on steel surfaces and in pipelines where food products are processed, establishing biofilms. Therefore, it is of utmost importance to find new treatments of surfaces and processing lines in the food industry in order to eliminate bacterial contaminations. Several approaches have been proposed to release of active ingredients to the surface and kill the micro-organisms. For example, Poverenov and colleagues prepared numerous active anti-microbial surfaces on the basis of polymers, cellulose, and glass, with potent inhibition against *Bacillus cereus* (*B. cereus*), *Alicyclobacillus acidoterrestris* (*A. acidoterrestris*), *Escherichia coli* (*E. coli*), and *Pseudomonas aeruginosa* (*P. aeruginosa*) [12,13,14,15,16]. Similar research on anti-microbial food-contact materials were developed based on curcumin [33,34,35], polyphenols and natural compounds [36,37,38], essential oils to control pest pathogens [39], active-passive modified atmosphere for microbial control [40,41], and various polymeric-based anti-microbial films [28,29,30,31,32]. Using nanotechnological approaches, new materials based on the antimicrobial property of silver nanoparticles have been studied and produced [42,43,44,45].

The perishable foods market is in the need of anti-microbial materials due to economic losses caused by bacterial and fungal growth on foods throughout the entire food supply chain. Such anti-microbial materials should extend the shelf-life of the product on the market shelves up to the consumer table. One challenge is to find methods for improved treatment (i.e., modified atmosphere, type of film, packages composed by various active materials) and application of effective, safe anti-bacterial and anti-fungal compounds [46,47,48,49,50,51,52]. These methods may ensure the safety of foods and alleviate the economic losses due to food deterioration. It is envisaged that new anti-microbial compounds could be incorporated in food packaging and films to improve the shelf-life of ready-to-eat foods and packaged fresh products.

In parallel to these studies, other recent reports described similar antibacterial properties in xanthones such as mangiferin from edible plants, as well as from other medicinal plants [53,54].

In the field of polymer preparation, electro-spinning techniques have been applied to *G. mangostana* extracts, with good results in formation of polylactic acid fiber mats to be used in wound dressing [31]. Electro-spinning allows the deposition of small and medium sized molecules on the surface of a forming polymer [31]. In addition, during the spinning and deposition of the bioactive compounds, eventual solvents present as residues of extraction or purification steps may be evaporated, reducing the possibility of contamination of material being in contact with food products. Furthermore, other deposition techniques may be applied with higher performance and improved purity of xanthones to become part of the polymer. Considering a covalent linkage of the bioactive core inside the polymer, a preliminary study examined functionalized polyxanthones in the form of poly-azoxanthone esters (PAXA), through polycondensation, and showed their applicability in food packaging and in pharmaceutical industry [32].

In search for new anti-bacterial agents we performed semi-synthetic modification of α-mangostin using Ritter reaction, reduction by palladium-carbon (Pd-C), alkylation and acetylation to improve the bioactivity of the base compound. In this study, we describe the selective enrichment of α-mangostin (demonstrated by the NMR peaks and HPLC graphs) its semi-synthetic modification, the products generated, their chemical structure, and the inhibition activity against four pathogens, two Gram-positive and two Gram-negative bacteria, and two fungi, evaluated as diameter or halo of growth inhibition. Herein, we studied the anti-microbial activity of α-mangostin and its synthetic analogs and confirmed the development of new anti-microbial xanthones with higher antibacterial and antifungal activity. The candidate molecules with higher bioactivity may be applied in the composition of antimicrobial textiles and polymers that could find applications in biomedical devices and in food packaging.

## 2. Results

### 2.1. α-Mangostin Isolation and Purification

α-Mangostin was isolated from the dried fruits of *G. mangostana* using ethyl acetate and yielded 26 g (26%) of dried ethyl acetate crude extract. The crude extract subjected to column chromatography with ethyl acetate and hexane yielded 5–6 g of pure α-mangostin and the purity was confirmed as 95% using HPLC. The structural characterization of the pure α-mangostin (**I**) using ^1^H-NMR spectra, ^13^C-NMR spectra, IR spectrum and high-resolution mass spectra is available in Supplementary materials.

### 2.2. Synthetic Modifications

**Figure 2:**
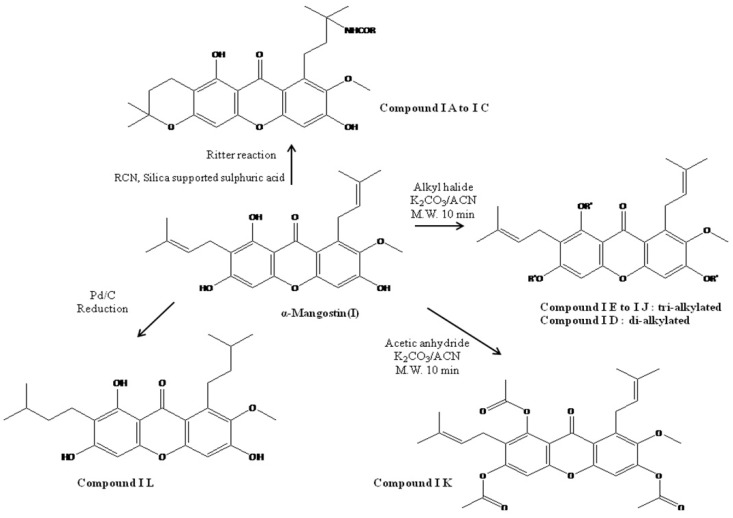
Reaction scheme leading to different derivatives.

Following the isolation, α-mangostin was subjected to a series of chemical reactions to alter the core structure (Figure 2).

The basic core structure xanthone (anthraquinone) was conserved intact while the functional iso-prenyl and phenolic hydroxy groups were subjected to semi-synthetic modification. Twelve different semi-synthetic derivatives were obtained (Table 1), each containing new key moieties that were evaluated to detect the anti-microbial activity against various bacterial and fungal cultures.

(**I D**), the alkylated product of α-mangostin with ethyl iodide, was found to be a di-alkylated product. All other alkylations using the exact same reaction conditions reported in the table resulted in tri-alkylated products. In the Ritter reaction, the addition of acetonitrile, followed by intra-molecular addition of –OH to isoprene unit gave ether (or) pyran ring structures. Addition of acetonitrile is followed by hydration to produce amide. This reaction is general for all the nitrile reaction products studied (**I A** to **I C**). Compound ID, reaction of α-mangostin with ethyl iodide, produced mainly the di-alkylated product, probably due to the possibility of ether cleavage of reaction product HI during the reaction. All other alkylation reactions carried out with alkyl-bromide yielded only tri-alkylated products (**I E** to **I J**).

### 2.3. Biological Assays

#### 2.3.1. Anti-Bacterial Assay

The evaluation of the anti-bacterial potential for α-mangostin-based synthetic analogs was examined against *E. coli*, *Bacillus subtilis* (*B. subtilis*), *S. aureus*, and *P. aeruginosa*, in accordance with an experimental procedure. Two different concentrations (50 μg/mL and 100 μg/mL) of the α-mangostin and their synthetic analogs along with standard drug Ciprofloxacin were tested against the pathogens and the results are given in Table 2. The zone of inhibition was determined in triplicates using the diffusion technique, with values representing the average zone of inhibition.

By measuring the zone of inhibition (in mm), it is observed that all the derivatives of α-mangostin exert moderate to high anti-bacterial activity. Compound (**I E**) showed maximum anti-bacterial activity (up to 12 mm) at 100 μg/mL concentration against all bacterial stains tested in comparison to the other synthesized compounds. At low concentration of 50 μg/mL, the acetyl derivative (**I G**) showed maximum inhibition against *E. coli*. The butyl derivative (**I C**) showed maximum inhibition against *B. subtilis*. The propenyl derivative (**I G**) showed maximum inhibition against *S. aureus*. The ethyl (**I D**) and benzene sulphonyl (**I I**) derivatives of α-mangostin showed maximum inhibition against *P. aeruginosa*. Among these compounds, the acetyl (**I K**) and benzene sulphonyl (**I I**) derivatives of α-mangostin showed maximum anti-bacterial activity against the four bacterial strains tested. Most of the derivatives showed better anti-bacterial activity against Gram-positive bacteria *B. subtilis* and *S. aureus* followed by the Gram-negative bacteria *P. aeruginosa* and *E. coli.* The agar disk-diffusion method may not be appropriate method to determine the minimum inhibitory concentration (MIC), as it is impossible to quantify the amount of the antimicrobial agent diffused into the agar medium. Since only two concentrations (50 and 100 μg/mL) were tested, at this time we could not calculate the appropriate MIC value. Further experiments are required to evaluate the effective microbicidal concentrations, such as MBC tests.

#### 2.3.2. Anti-Fungal Assay

The first compound evaluated was the natural product α-mangostin, which was compared against the synthetic analogs to prove that the analogs had better efficacy than the parent molecule. Two compounds, **I H** and **I J**, at 100 µg/mL concentration, showed maximum anti-fungal activity against *Candida albicans*, with a 13 mm inhibition halo, in respect to the other tested compounds. In addition, among all derivatives of α-mangostin, the acetyl (**I K**) and benzene sulphonyl (**I I**) derivatives at 100 μg/mL concentration displayed maximum inhibition of 13 mm against *Aspergillus niger* (Table 2). The zone of inhibition was determined in triplicates using the diffusion technique, with values representing the average zone of inhibition. The alkylated product of α-mangostin (**I I**) showed the most significant activity against fungal strains, with 12 mm inhibition of *C. albicans* and 13 mm inhibition of *A. niger*. The xanthonoid skeleton with benzene sulphonyl moiety showed enhanced anti-fungal activity for α-mangostin-based derivatives.

The cytotoxicity studies were carried out in silico using a software to individuate reactive groups as from databases on chemical compounds. The in silico toxicity was predicted using the Toxtree implementation of the modified Cramer rules and Verhaar scheme. The software Toxtree v.2.6.13 (IdeaConsult Ltd., Sofia, Bulgaria), along with Cramer rules and Verhaar scheme, were run to study the toxicity of organic molecules [55,56,57,58,59]. The module was developed for the Long-Range Research Initiative (LRI) and European Chemical Industry Council (CEFIC) CEFIC-LRI project AMBIT for a new open software tool. A typical evaluation is given for one of the products. The study showed all the molecules to be non-toxic. However this has to be validated by in vitro or in vivo studies which are beyond the scope of the present study. Indeed such studies will form part of an in depth evaluation of potential molecules. The in silico studies for compounds **I A**–**C** are attached as separate file named “Mangostin and derivatives IA-IC in silico studies”. An example of results of cytotoxicity studies is presented in the Supplementary file S2, “**I A**, **I B**, **I C** α-mangostin derivatives in silico toxicity test”.

## 3. Discussion

In this study the semi-synthetically modified α-mangostin-derived compounds were shown to exhibit bioactive properties and antimicrobial activity greater than the parent natural molecule when tested against various microbial and fungal cultures. Due to their anti-microbial properties, they can be utilized as wound healers and in the treatment of skin infections, and in packaging materials for the extension of food shelf life. We have shown the semi-synthetic modification of α-mangostin using the Ritter reaction reduction by palladium-carbon (Pd-C), alkylation, and acetylation.

Studies are ongoing in the field of materials for packaging, to extend the shelf-life of food products. The biomedical industry is also interested in a safe way for the containment of biofilm formation, and production of devices with potential to avoid bacterial growth. Textiles, polymer films, and wrapping materials are manufactured by electro-spinning and layer-by-layer deposition, the first one better suited to eliminate residual solvents. The perishable foods represent a loss due to fungal and bacterial growth, requiring short times from the shelves to the table. Thus, improvement in treatments and use of anti-fungal films may ensure higher safety standard for human health in addition to decrease of economic losses at retailer shops and during food supply chain. It is expected that new materials and films, based on available and novel, semi-synthetic antimicrobial compounds, after assessment of safety for humans or animals, grade of release of antimicrobials during the time, may find their application according to the industry needs.

α-Mangostin and derived semi-synthesis products have been shown in several studies to have the potential to be exploited as antimicrobial agents. In this study, twelve α-mangostin derivatives were analyzed for inhibition of two Gram-positive and two Gram-negative bacteria (using Ciprofloxacin as a reference drug at 50 and 100 µg/mL) and two fungal pathogens (using Ketoconazol as a reference drug at 50 and 100 µg/mL) to determine the sensitivity of each bacterial species. 

Looking for derivatives with improved activity in respect to the original compound α-mangostin xanthone. Starting from this preliminary characterization, it will be possible to test these compounds in vitro and in vivo, on animal models, to check the safety of the compounds, and to evaluate the inhibition of bacterial growth, such as a bactericide activity on infected wounds. The antimicrobial compounds, such as **I E,** have been shown to have inhibitory activity and need to be studied further for applications in healthcare.

## 4. Materials and Methods

### 4.1. Isolation and Purification of α-Mangostin from Mangoosteen Fruit

All of the chemicals and reagents were purchased from either Sigma-Aldrich (St. Louis, MO, USA) or Merck (Darmstadt, Germany) chemicals. The extraction of α-mangostin from the dried fruits of *G. mangostana* was carried out with Soxhlet extraction methodology. A known weight (100 g) of the fruit hulls was used, and extracted twice with 300 mL of ethyl acetate. The extract was filtered through a Whatman filter paper No.1 (Brentford, UK) by suction. The filtrate was concentrated under reduced pressure to obtain the ethyl acetate crude extract (26 g). Then 20 g of ethyl acetate crude extract was subjected to TLC column chromatography using ethyl acetate and hexane (1:1) as the eluent. The fractions containing α-mangostin were pooled and evaporated to obtain a solid of 95% purity by HPLC (High Performance Liquid Chromatography). The obtained solid fraction was used as starting material for semi-synthetic modification and for microbial studies. The compound was repeatedly recrystallized using benzene for structural characterization using ^1^H-NMR spectra, ^13^C-NMR spectra, and high-resolution mass spectra. 

### 4.2. General Methods for Compound Analysis

The purity of the isolated α-mangostin and the progress of the reaction was monitored by HPLC on analytical reversed phase develosil ODS column C18 (150 mm × 4.6 mm, 0.5 µm) using 0.02 M potassium dihydrogen phosphate in water and a acetonitrile 50:50 ratio as the mobile phase, with 1.0 mL/min flow rate for 30 min, and a UV detector wavelength of 254 nm. The main product was analyzed by nuclear magnetic resonance (NMR) (^1^H: 400 MHz, ^13^C: 100 MHz) and data recorded on a Bruker instrument (Billerica, MA, USA), with chemical shifts expressed in δ ppm. NMR spectra were obtained in MeOD with tetramethylsilane (TMS) as a reference compound. Mass was determined using a Shimadzu analyzer (Columbia, MD, USA).

### 4.3. Anti-Microbial Activity Assay

The study of anti-microbial activity of the synthetic analogs was performed by measuring the diameter of the inhibition halo from the outer surface of the disc, expressed in millimeters. The zone of inhibition of the α-mangostin was compared with its synthetic analogs to determine the rate of inhibition. The zone of inhibition was determined in triplicates using the diffusion technique, with values representing the average zone of inhibition. The nutrient broth medium (50 mL) was prepared and sterilized in autoclave at 121 °C for 15 min. Gram-positive bacteria (*B. subtilis* and *S. aureus*) and Gram-negative bacteria (*E. coli* and *P. aeruginosa*) were inoculated in tubes of nutrient broth, whereas the fungal cultures (*C. albicans* and *A. niger*) were inoculated in tubes of potato dextrose agar, and incubated at 37 °C for 24 h; then the suspension was centrifuged at 8000× *g* for 5 min, the pellet was suspended in double-distilled water, and the cell density was standardized spectrophotometrically (A_610_ nm). All of the microbial cultures were adjusted to 0.5 McFarland standards, which is visually comparable to a microbial suspension of approximately 1.5 × 10^8^ cfu/mL.

#### 4.3.1. Anti-Bacterial Assay

The bacterial species, chosen as representatives of spoilage and pathogenic species commonly found contaminating surfaces and workplaces, were used to evaluate the anti-bacterial activities of the synthetic compounds. The bacteria were maintained on Muller Hilton broth media at 37 °C. Then, Muller Hilton agar plates were swabbed with 100 μL inocula of the test microorganisms and kept for 15 min for absorption. Six-millimeter Whatman No. 1 discs were pre-sterilized and two concentrations (50 and 100 μg/mL) of the test compounds in DMSO were applied to the sterile disc papers. The standard drug Ciprofloxacin (50 and 100 μg) was used as a positive reference standard to determine the sensitivity of each bacterial species. Then the plates were inoculated at 37 °C for 24 h.

#### 4.3.2. Anti-Fungal Assay

All of the synthesized compounds were screened for anti-fungal activity by disc diffusion method. PDA medium was autoclaved at 121 °C for 15 min and poured into each Petri plate and the solidified media plates were swabbed with 0.2 mL of fungal cultures. Six-millimeter Whatman No. 1 discs were loaded with the test compounds in DMSO at 50 and 100 μg/mL concentration. Then the plates were inoculated at 28 °C for 72 h. The diameter of the clear zone around the well was measured and expressed in millimeters. Standard drug Ketoconazole (50 and 100 μg) was used as a positive reference standard to determine the sensitivity of each fungal species.

## 5. Conclusions

Among the semi-synthetic derivatives of α-mangostin, **I E** showed higher anti-bacterial activity whereas **I I** showed the most significant anti-fungal activity. These compounds pave the way to the synthesis of non-toxic compounds with better efficacy compared to the natural product for anti-microbial treatment. It is envisaged that these new anti-microbial compounds could be incorporated in packaging materials, textiles, biomedical devices, and film polymers, to improve the microbiological safety of surfaces in contact with bacteria.

## Figures and Tables

**Table 1:** α-Mangostin and its synthetic derivatives.

S. No.	Compound Information	Structure	Reaction of α-Mangostin with	Molecular Formula	Mass
1.	α-Mangostin **(I)**	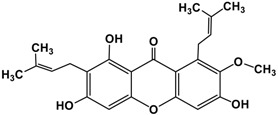	-	C_24_H_26_O_6_	411 (M+1)^+^
2.	Ritter product of α-mangostin **(I A)**	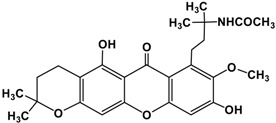	Acetonitrile in Silica supported sulphuric acid	C_26_H_31_NO_7_	470 (M+1)^+^
3.	Ritter product of α-mangostin **(I B)**	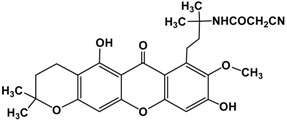	Malononitrile in Silica supported sulphuric acid	C_27_H_30_N_2_O_7_	514 (M+2+NH_3_)^+^
4.	Ritter product of α-mangostin **(I C)**	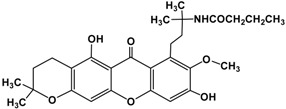	Butyronitrile in Silica supported sulphuric acid	C_28_H_25_NO_7_	498 (M+1)^+^
5.	Alkylated product of α-mangostin **(I D)**	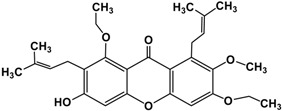	Ethyl Iodide	C_28_H_34_O_6_	467 (M+1)^+^
			K_2_CO_3_/ACN		
			M.W. 10 min		
6.	Alkylated product of α-mangostin **(I E)**	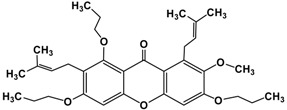	Bromopropane	C_33_H_44_O_6_	537 (M)^+^
			K_2_CO_3_/ACN		
			M.W. 10 min		
7.	Alkylated product of α-mangostin **(I F)**	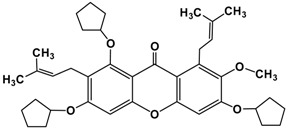	Cyclopentyl bromide	C_39_H_50_O_6_	615 (M)^+^
			K_2_CO_3_/ACN		
			M.W. 10 min		
8.	Alkylated product of α-mangostin **(I G)**	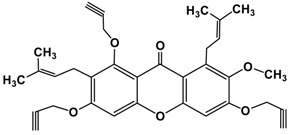	Propargyl bromide	C_33_H_32_O_6_	525 (M)^+^
			K_2_CO_3_/ACN		
			M.W. 10 min		
9.	Alkylated product of α-mangostin **(I H)**	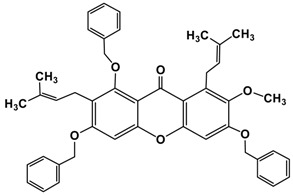	Benzyl bromide	C_45_H_44_O_6_	681 (M)^+^
			K_2_CO_3_/ACN		
			M.W. 10 min		
10.	Alkylated product of α-mangostin **(I I)**	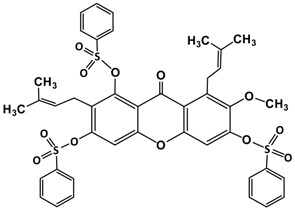	Benzene sulphonyl chloride	C_42_H_38_O_12_S_3_	831 (M)^+^
			K_2_CO_3_/ACN		
			M.W. 10 min		
11.	Alkylated product of α-mangostin **(I J)**	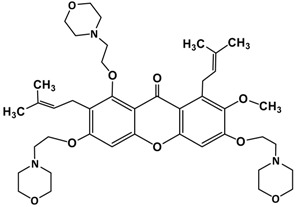	Chloroethyl morpholine hydrochloride	C_42_H_59_N_3_O_9_	750 (M)^+^
			K_2_CO_3_/ACN		
			M.W. 10 min		
12.	Acylated product of α-mangostin **(I K)**	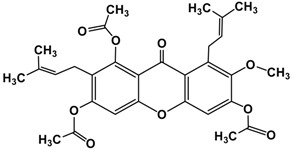	Acetic anhydride	C_26_H_28_O_7_	536 (M)^+^
			K_2_CO_3_/ACN		
			M.W. 10 min		
13.	Reduced product of α-mangostin **(I L)**	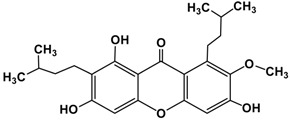	Palladium/Carbon	C_25_H_30_O_6_	415 (M+1)^+^

**Table 2:** Anti-microbial and anti-fungal activity of the α-mangostin and their synthetic analogs.

S. No.	Zone of Inhibition in mm											
	*E. coli*		*B. subtilis*		*S. aureus*		*P. aeruginosa*		*C. albicans*		*A. niger*	
Conc. (μg/mL)	50	100	50	100	50	100	50	100	50	100	50	100
**I**	5 ± 0.09	8 ± 0.04	7 ± 0.11	9 ± 0.15	5 ± 0.05	9 ± 0.12	6 ± 0.24	10 ± 0.08	8 ± 0.12	10 ± 0.14	4 ± 0.18	7 ± 0.05
**I A**	4 ± 0.18	9 ± 0.16	6 ± 0.15	10 ± 0.11	5 ± 0.12	11 ± 0.22	5 ± 0.11	8 ± 0.16	7 ± 0.11	10 ± 0.06	9 ± 0.11	12 ± 0.02
**I B**	6 ± 0.14	10 ± 0.11	6 ± 0.12	9 ± 0.23	5 ± 0.16	8 ± 0.14	4 ± 0.08	8 ± 0.22	4 ± 0.16	6 ± 0.11	3 ± 0.07	5 ± 0.15
**I C**	8 ± 0.11	11 ± 0.08	10 ± 0.06	12 ± 0.04	6 ± 0.18	9 ± 0.06	5 ± 0.16	10 ± 0.13	2 ± 0.16	6 ± 0.13	2 ± 0.18	5 ± 0.14
**I D**	7 ± 0.12	10 ± 0.18	4 ± 0.04	10 ± 0.09	5 ± 0.08	7 ± 0.12	9 ± 0.21	9 ± 0.08	4 ± 0.22	6 ± 0.21	7 ± 0.15	9 ± 0.08
**I E**	6 ± 0.13	11 ± 0.22	9 ± 0.15	12 ± 0.16	7 ± 0.22	11 ± 0.18	7 ± 0.05	12 ± 0.03	4 ± 0.08	7 ± 0.17	3 ± 0.04	5 ± 0.05
**I F**	6 ± 0.18	9 ± 0.04	5 ± 0.09	11 ± 0.18	7 ± 0.14	11 ± 0.17	5 ± 0.09	9 ± 0.14	3 ± 0.07	5 ± 0.14	4 ± 0.09	7 ± 0.11
**I G**	6 ± 0.20	9 ± 0.12	7 ± 0.05	10 ± 0.11	10 ± 0.21	11 ± 0.19	6 ± 0.14	9 ± 0.18	4 ± 0.12	6 ± 0.19	3 ± 0.16	5 ± 0.19
**I H**	5 ± 0.12	9 ± 0.11	6 ± 0.12	9 ± 0.08	8 ± 0.18	10 ± 0.13	8 ± 0.11	11 ± 0.11	9 ± 0.17	13 ± 0.12	7 ± 0.11	10 ± 0.21
**I I**	5 ± 0.08	8 ± 0.15	6 ± 0.04	10 ± 0.20	7 ± 0.11	9 ± 0.15	9 ± 0.19	12 ± 0.06	10 ± 0.14	12 ± 0.09	9 ± 0.08	13 ± 0.11
**I J**	4 ± 0.15	7 ± 0.16	5 ± 0.11	8 ± 0.14	4 ± 0.13	7 ± 0.21	3 ± 0.08	6 ± 0.20	11 ± 0.12	13 ± 0.15	6 ± 0.03	8 ± 0.18
**I K**	10 ± 0.14	12 ± 0.16	4 ± 0.10	7 ± 0.14	5 ± 0.08	9 ± 0.08	6 ± 0.22	9 ± 0.06	8 ± 0.20	11 ± 0.18	10 ± 0.15	13 ± 0.18
**I L**	9 ± 0.13	11 ± 0.06	5 ± 0.21	8 ± 0.16	5 ± 0.12	8 ± 0.05	6 ± 0.15	8 ± 0.12	7 ± 0.13	10 ± 0.08	9 ± 0.17	12 ± 0.08
**Std drug**	19 ± 0.11	24 ± 0.05	16 ± 0.07	20 ± 0.14	11 ± 0.15	18 ± 0.06	15 ± 0.12	19 ± 0.16	15 ± 0.16	18 ± 0.04	14 ± 0.13	18 ± 0.08

## Supplementary Materials

The following are available online. Figure S1: title Molecules Narasimhan Poltronieri supplementary.doc in Supplementary.zip. Supplementary materials include ^1^H-NMR spectra, ^13^C-NMR spectra, and high-resolution mass spectra for compounds **I**, **I A**–**I L**. Supplementary materials can be accessed from the PhD thesis “Studies on isolation and the semi-synthetic modification of bio active molecules from *G. mangostana* and *Embelia ribes*” submitted in partial fulfillment of the requirements for the degree of Doctor of Philosophy by S. Maheshwaran, under the guidance of Dr. S. Narasimhan at the University of Madras (April 2013). Supplementary word file S2: “**I A**, **I B**, **I C** α-mangostin derivatives in silico toxicity test”.
