# Supplementary material for: Anti-Bacterial and Anti-Fungal Activity of Xanthones Obtained via Semi-Synthetic Modification of α-Mangostin from Garcinia mangostana
Source: Molecules. 2017 Feb 12;22(2):275. doi: 10.3390/molecules22020275 (PMC6155947; doi:10.3390/molecules22020275)
Supplement: Supplementary file 1 [file molecules-22-00275-s001.pdf]

Supplementary Materials: Anti-Bacterial and Anti-Fungal Activity of Xanthones Obtained via Semi-Synthetic Modification of α-Mangostin from *Garcinia mangostana*

Narasimhan Srinivasan, Maheshwaran Shanmugam, Imad A. Abu-Yousef, Amin F. Majdalawieh, Rethavathi Janarthanam, Prince Edwin Das and Palmiro Poltronieri

^{1}H-NMR spectra, ^{13}C-NMR spectra, and high-resolution mass spectra for compounds (I, I A to I L)

1. MANGOSTIN (I)

Fig 1a: HPLC Chromatogram of standard, Standard purity plot and UV max

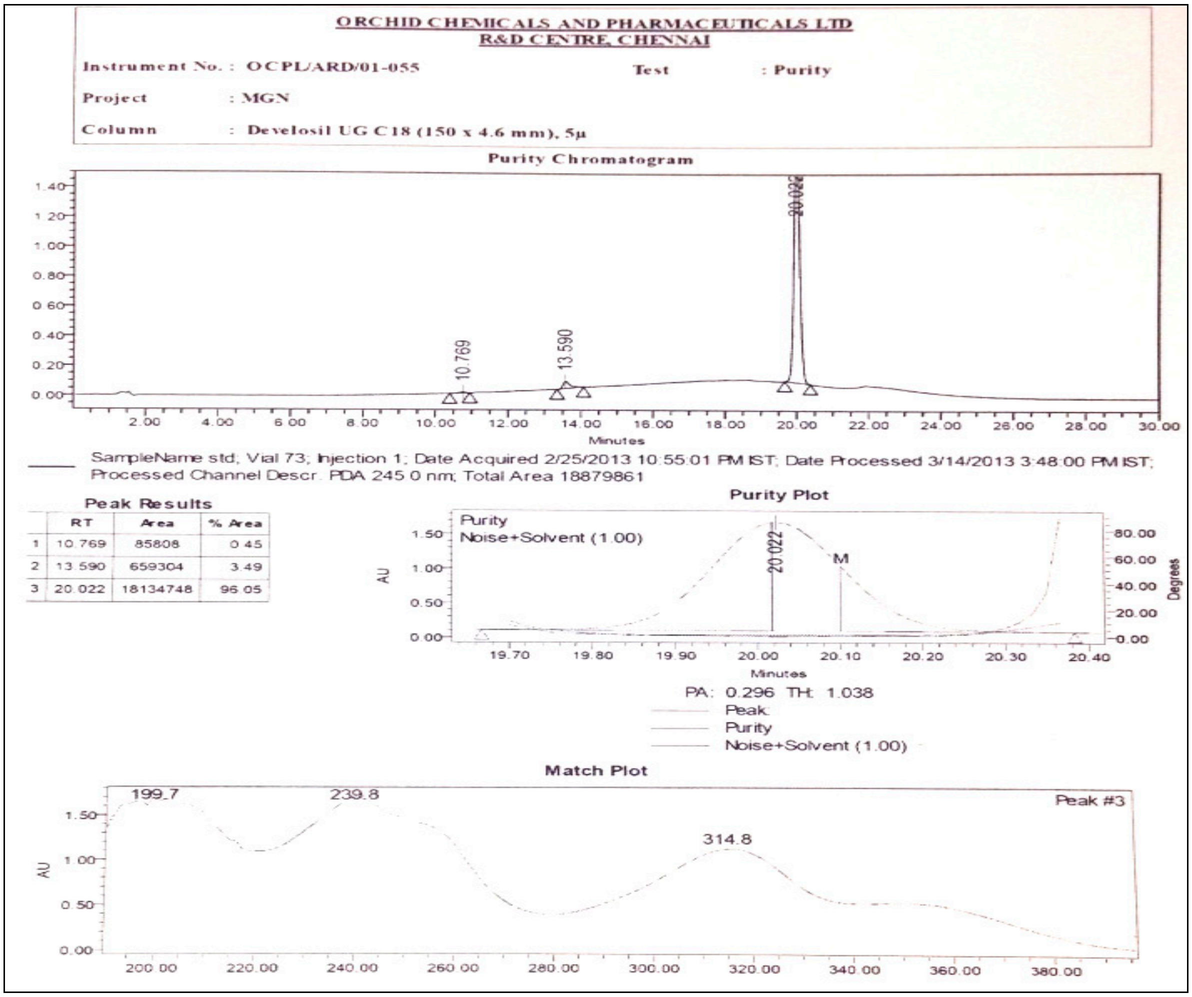

Fig 1b: ^{1}H NMR Spectrum of (I)

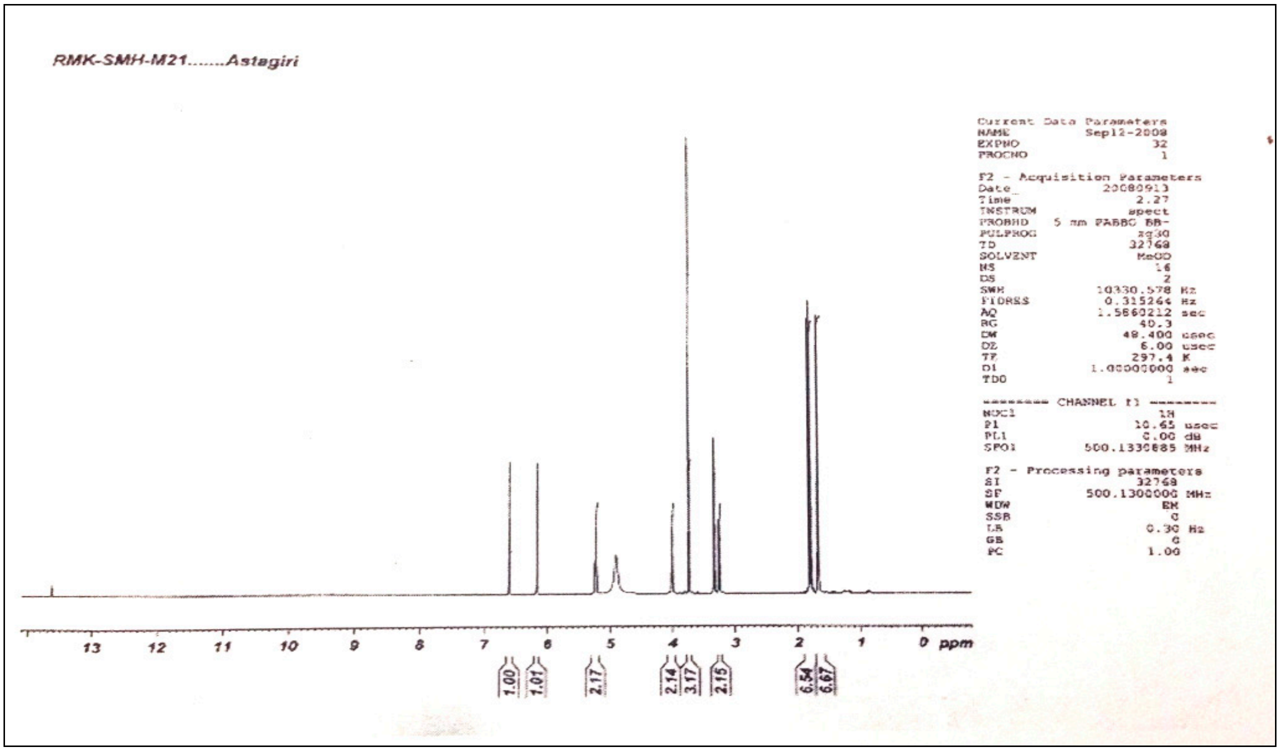

Fig 1c: ^{13}C NMR Spectrum of (I)

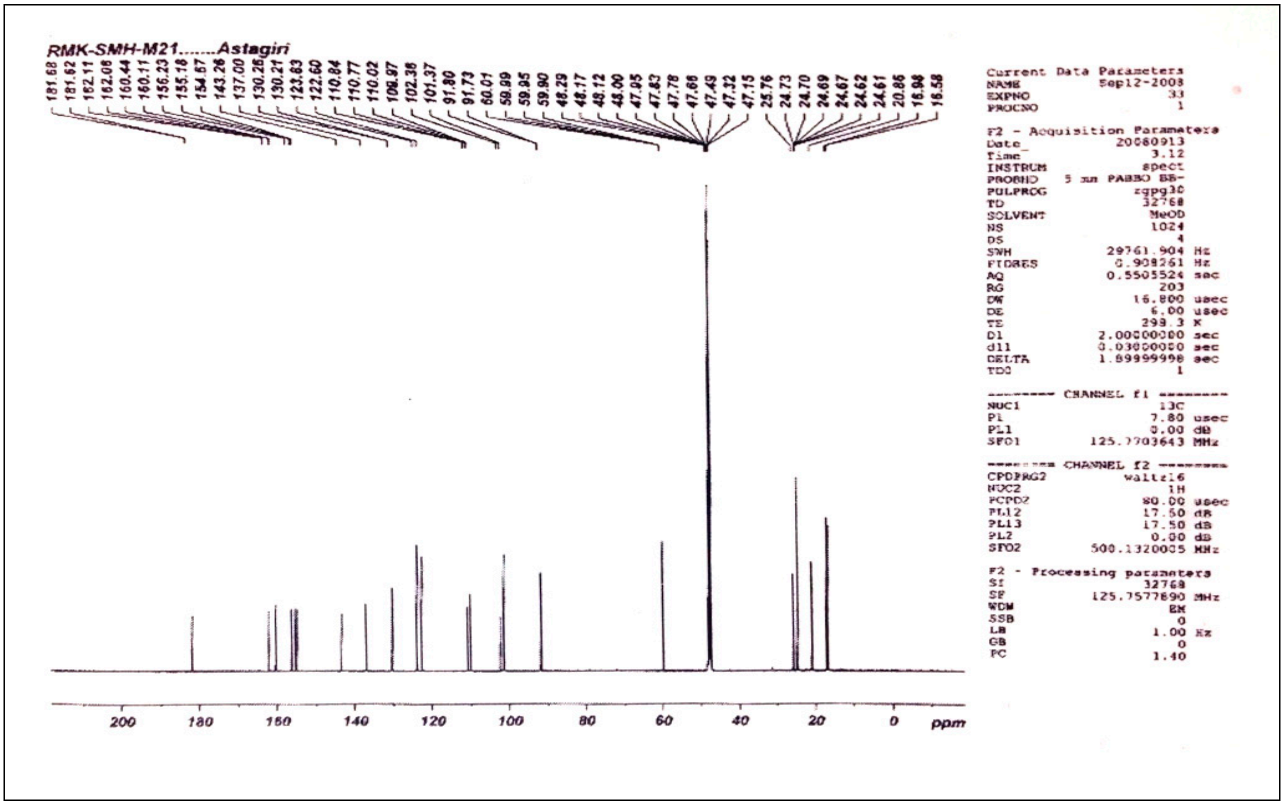

Fig 1d: IR Spectrum of (I)

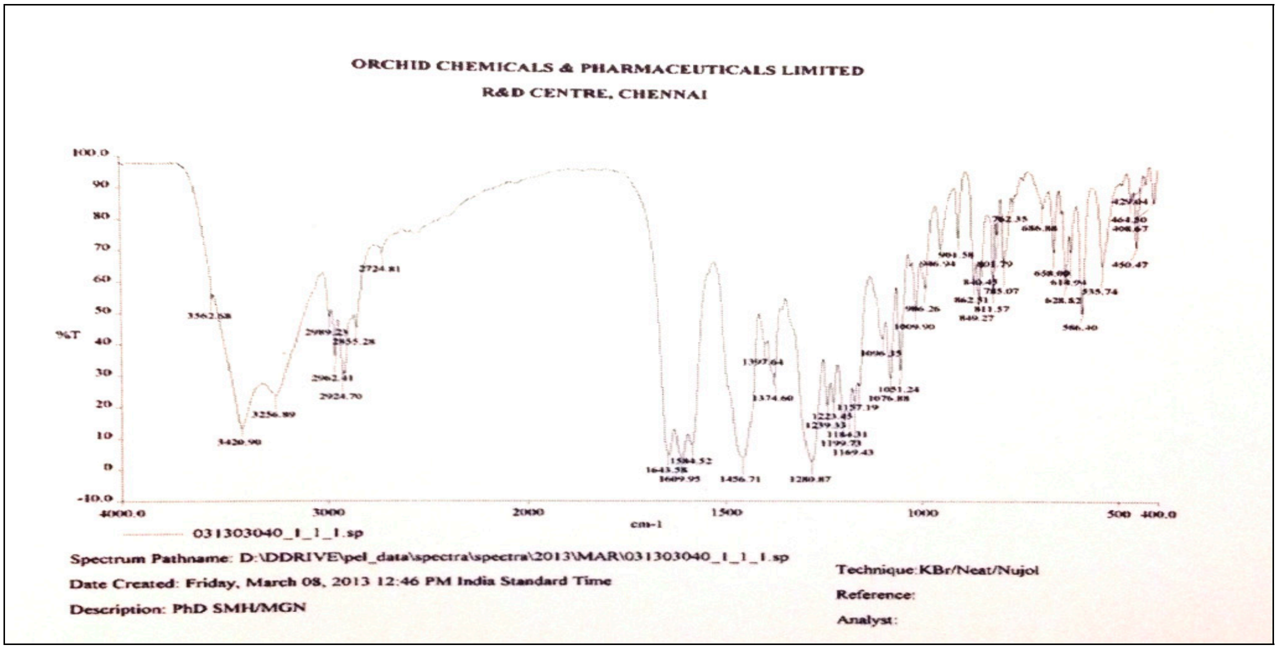

Fig 1b: Mass Spectrum of (I)

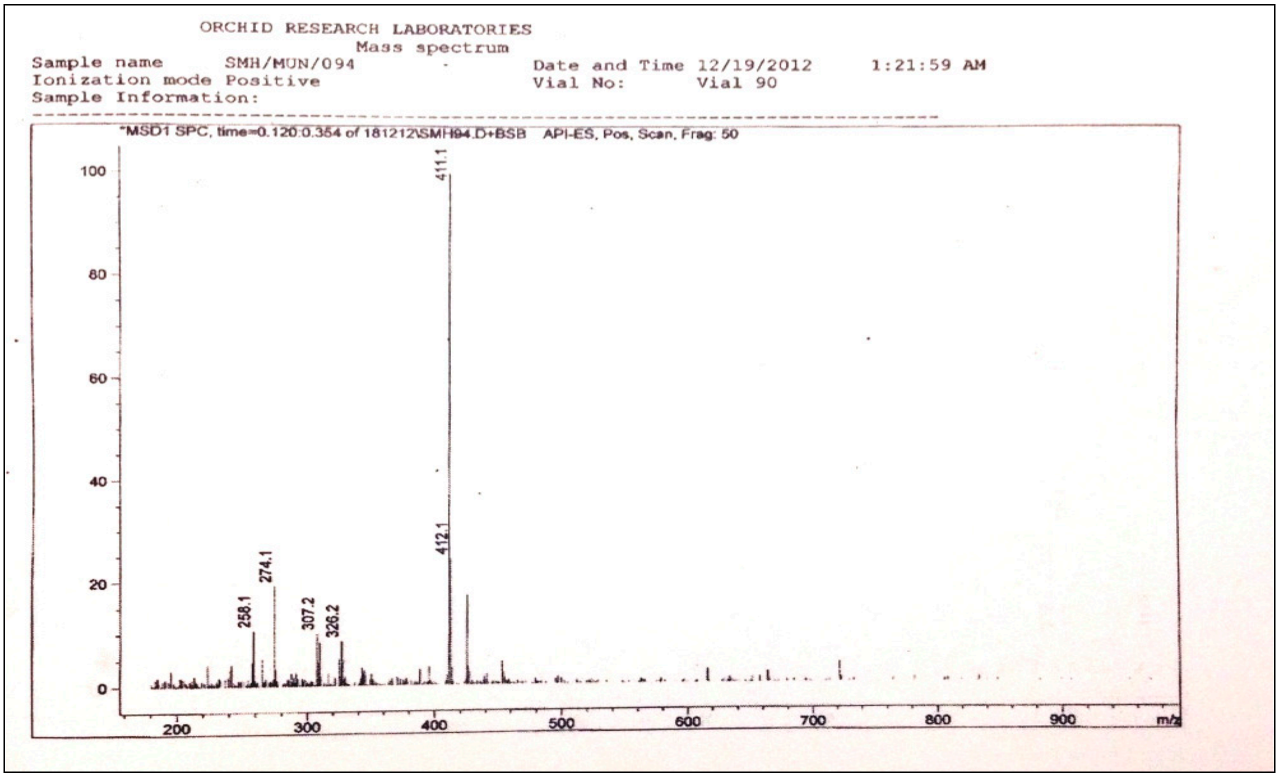

Table 1: NMR spectral Data of (I)
NMR: 400MHz (solvent: CDCl3, δ, ppm), ppm: parts per million

Carbon	13C NMR (δ ppm)	^{1}H NMR
1	156.23	-
2	109.9	-
3	162.1	-
4	91.8	6.60(s,1H)
5	101.3	6.16(s,1H)
6	155.18	-
7	143.2	-
8	137.0	-
9	181.6	-
10	102.3	-
11	154.67	-
12	110.8	-
13	155.18	-
14	20.8	4.01 (d,2H,J=6.3Hz)
15	122.6	5.22 (t,1H,J=6.3Hz)
16	130.2	-
17	24.7	1.77(s,3H)
18	16.5	1.81 (s,3H)
19	25.7	3.33(d,2H,J=7.1Hz)
20	123.8	5.26(t,1H,J=7.1Hz)
21	130.26	-
22	24.73	1.77 (s,3H)
23	16.9	1.81 (s,3H)
OCH_3	60.0	3.73(s,3H)

2. Ritter product of α-Mangostin (IA)

Fig 2a: Mass Spectrum of (I A)

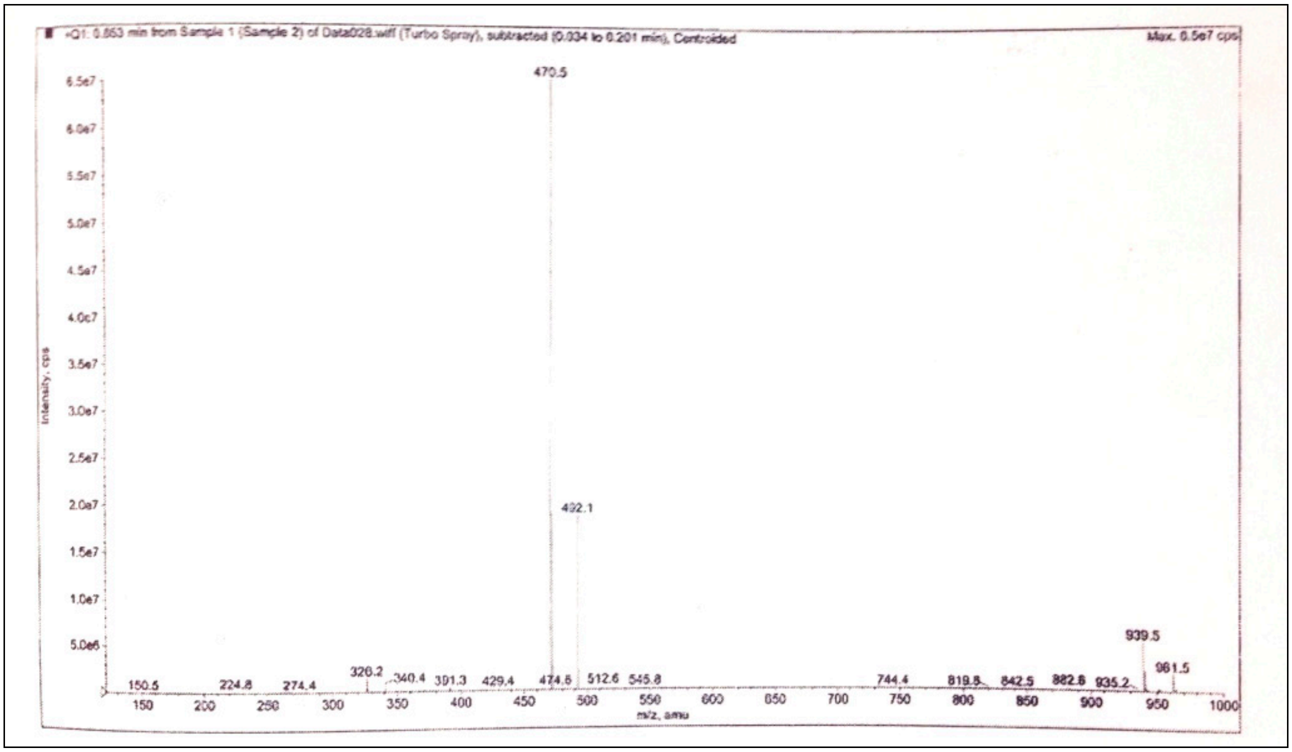

Fig 2b: ^{1}H NMR Spectrum of (I A)

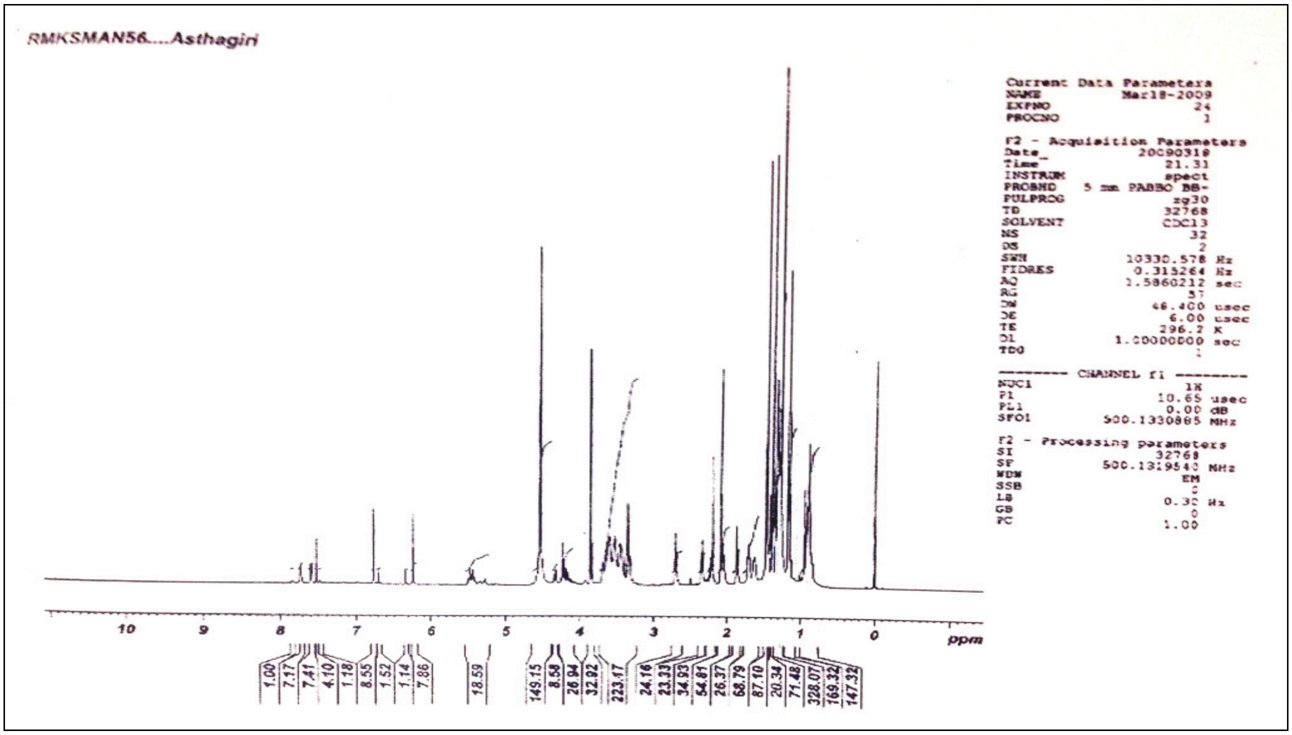

Fig 2c: ^{13}C NMR Spectrum of (I A)

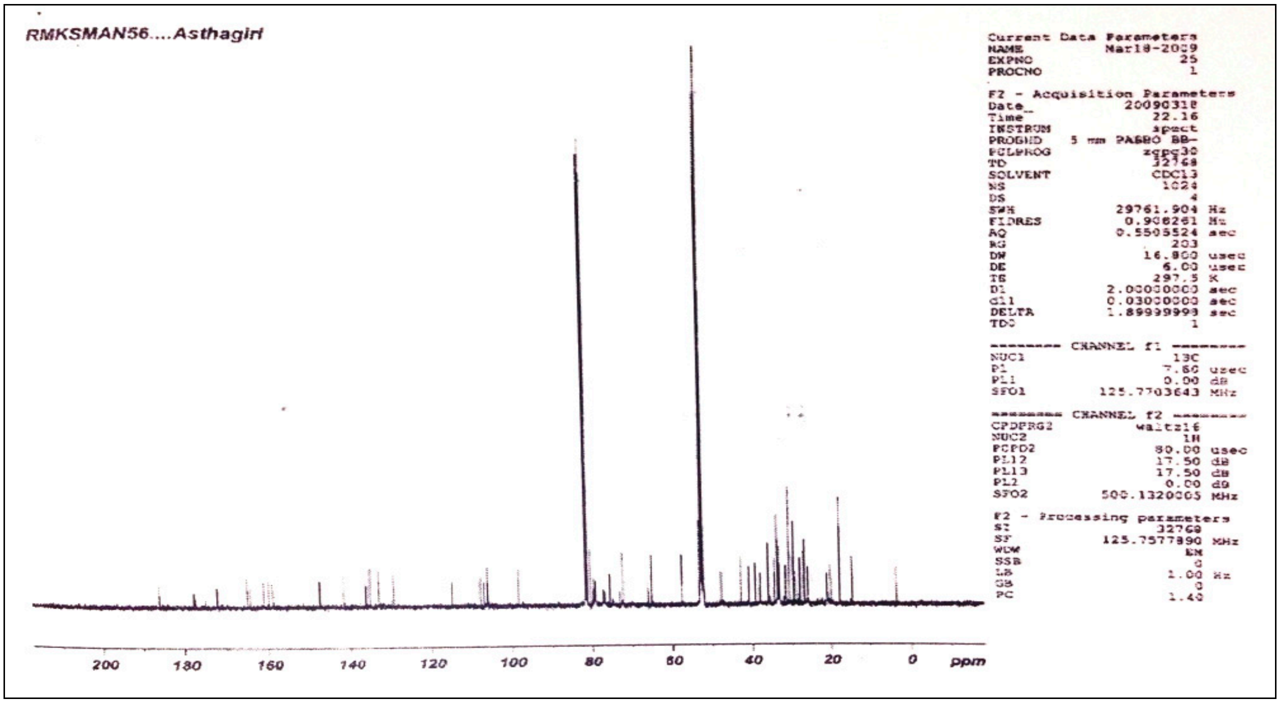

Table 2: NMR Spectral data of (I A)

NMR: 500MHz (Solvent: $CDCl_3$, δ, ppm), ppm: parts per million

Atom	^{13}C NMR (δ ppm)	^{1}H NMR (δ ppm)
1	160.79	-
2	107.65	-
3	164.86	-
4	98.02	6.76(s,1H)
5	105.94	6.24(s,1H)
6	163.97	-
7	147.33	-
8	141.45	-
9	186.17	-
10	106.57	-
11	158.74	-
12	114.58	-
13	159.85	-
14	17.67	2.34(t,2H,J=75.5Hz)
15	47.57	2.70(t,2H,J=6.5Hz)
16	57.35	
17	26.43	1.46(s,3H)
18	26.46	1.46(s,3H)
19	29.19	3.35(s,2H)
20	30.30	1.72(m,2H)
21	80.08	-
22	33.40	1.26(s,3H)
23	33.48	1.26(s,3H)
-NH$\underline{CO}$CH$_3$	172.12	-
CO$\underline{CH_3}$	25.66	2.08(s,3H)
$\underline{OCH_3}$	64.95	3.85(s,3H)

3. Ritter product of α-Mangostin (I B)

Fig 3a: Mass Spectrum of (I B)

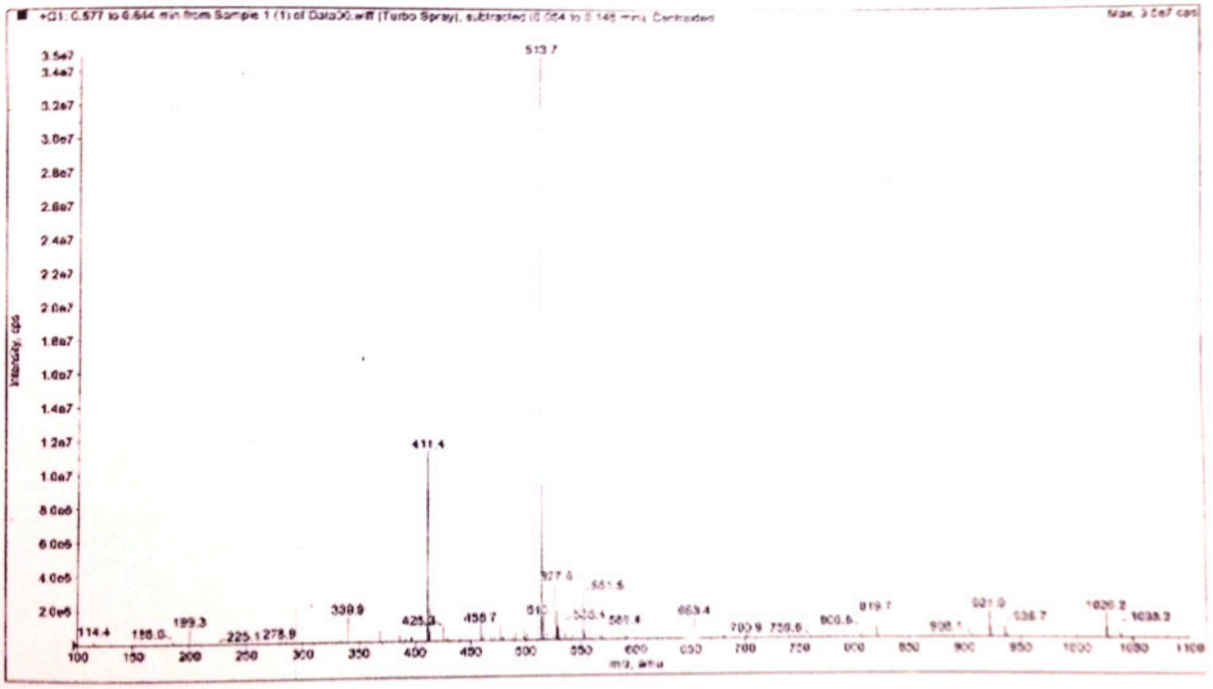

Fig 3b: ^{1}H NMR Spectrum of (I B)

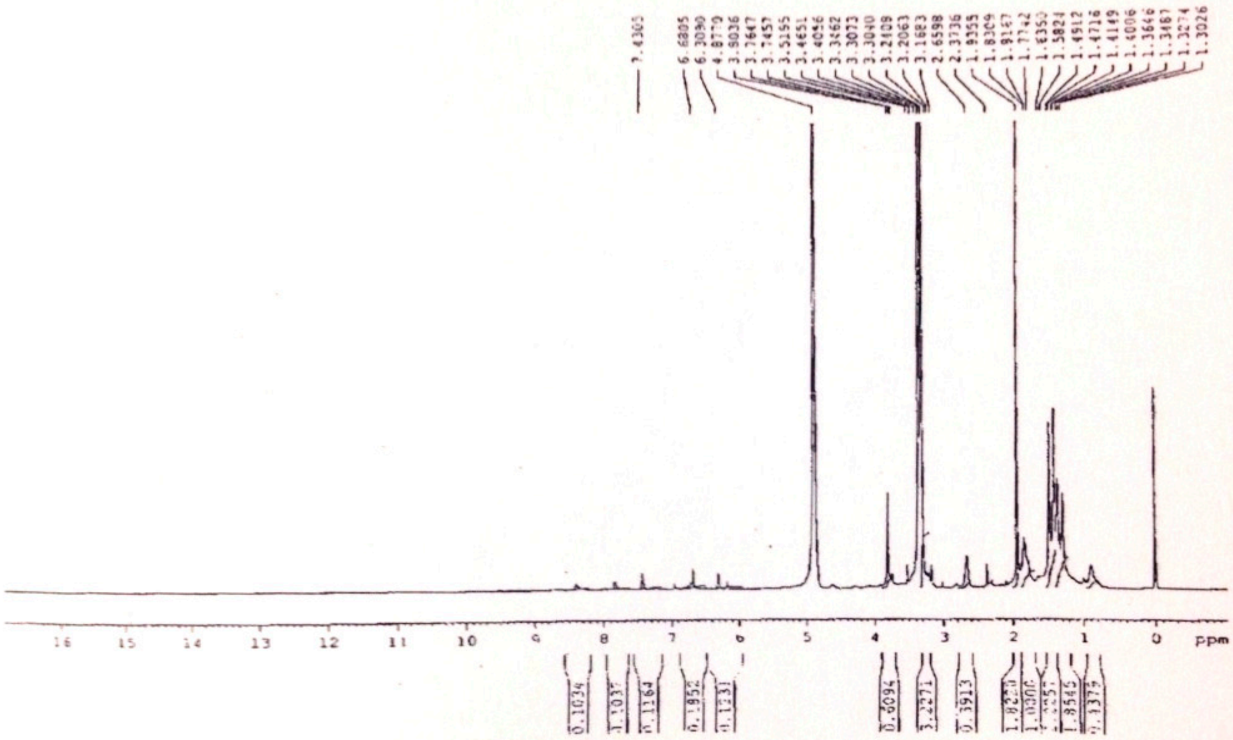

Fig 3c: ^{13}C NMR Spectrum of (I B)

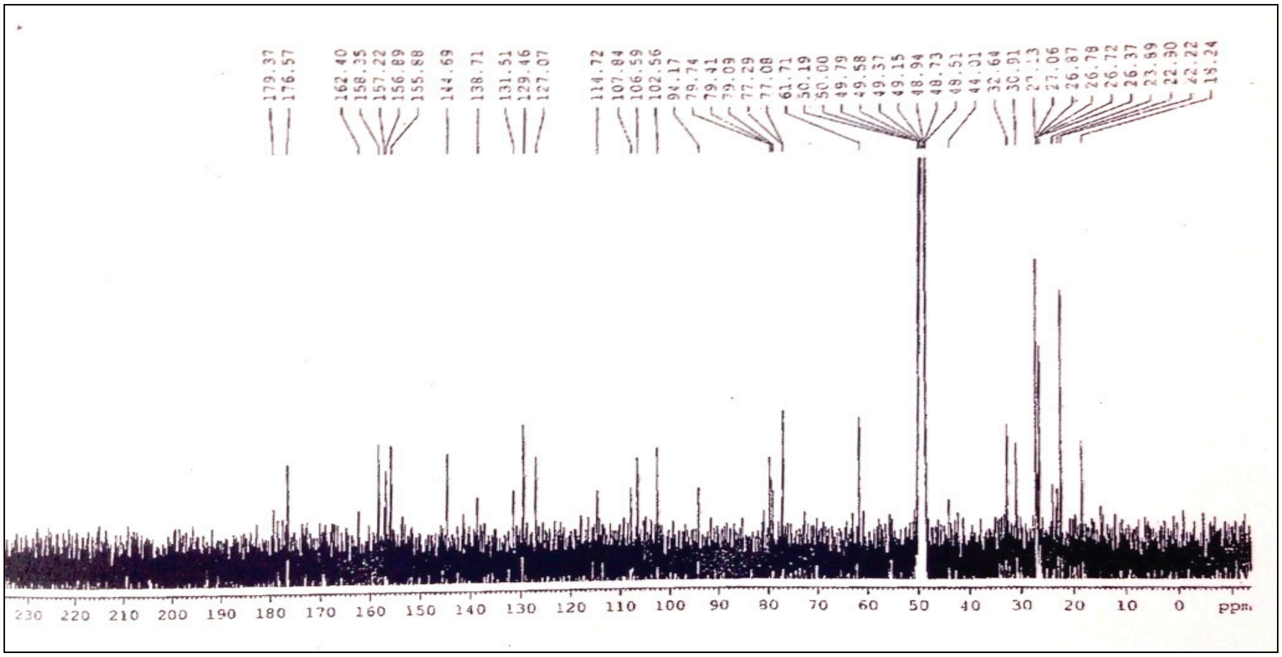

Table 3: NMR Spectral data of (I B)

NMR: 400MHz (Solvent: CD_3OD, δ, ppm), ppm: parts per million

Atom	^{13}C NMR (δ ppm)	^{1}H NMR (δ ppm)
1	157.22	-
2	107.84	-
3	162.40	-
4	94.17	6.68(s,1H)
5	102.56	6.30(s,1H)
6	158.35	-
7	144.69	-
8	138.71	-
9	179.37	-
10	106.59	-
11	155.88	-
12	114.58	-
13	156.89	-
14	18.24	2.37 (t, 2H, J=7.5Hz)
15	44.01	2.65 (t, 2H, J=6.5Hz)
16	50.19	-
17	27.13	1.58 (s, 3H)
18	27.13	1.58 (s, 3H)
19	30.91	3.34 (s, 2H)
20	32.64	1.77 (m, 2H)
21	77.29	-
22	26.37	1.49 (s, 3H)
23	26.37	1.49 (s, 3H)
$-NH\underline{CO}CH_3$	176.57	-
$CO\underline{CH_2}CN$	22.90	1.93 (s, 3H)
$COCH_2CN$	127.07	-
$\underline{OCH_3}$	61.71	3.80 (s, 3H)

4. Ritter product of α-Mangostin (I C)

Fig 4a: Mass Spectrum of (I C)

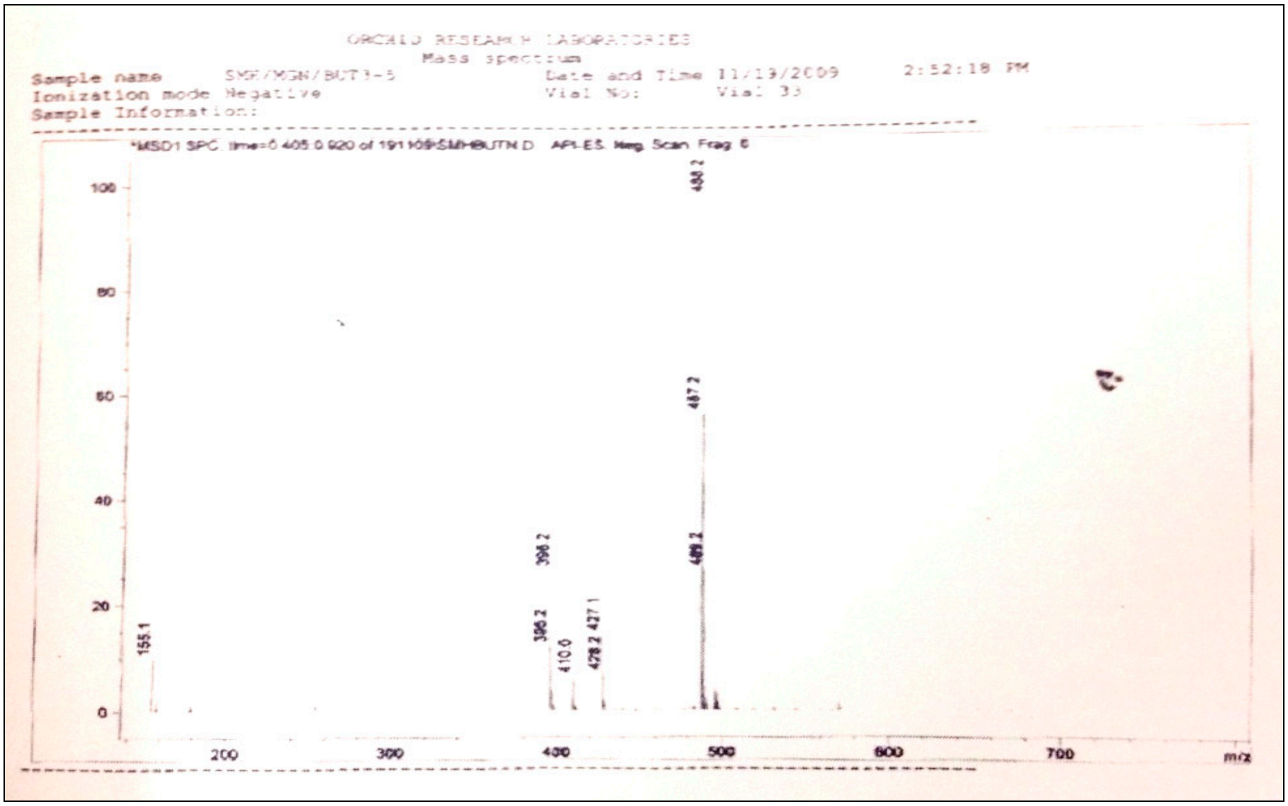

Fig 4b: ^{1}H NMR Spectrum of (I C)

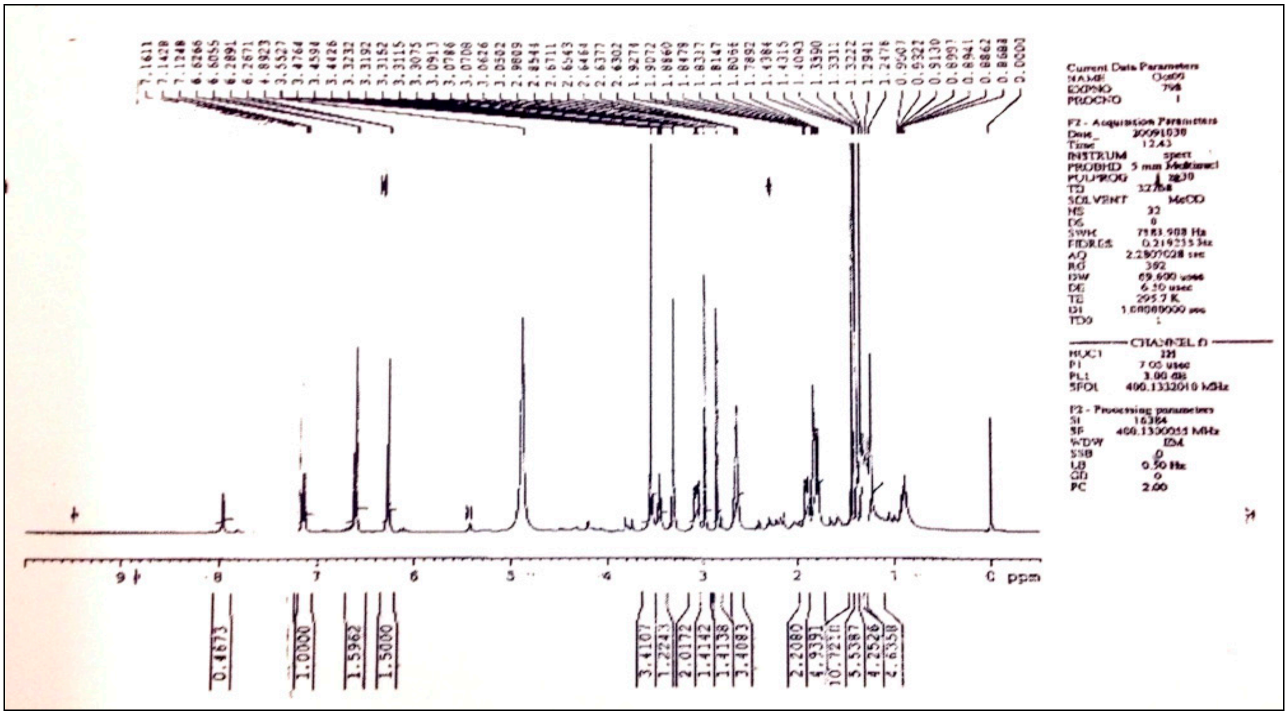

Fig 4c: ^{13} C NMR Spectrum of (I C)

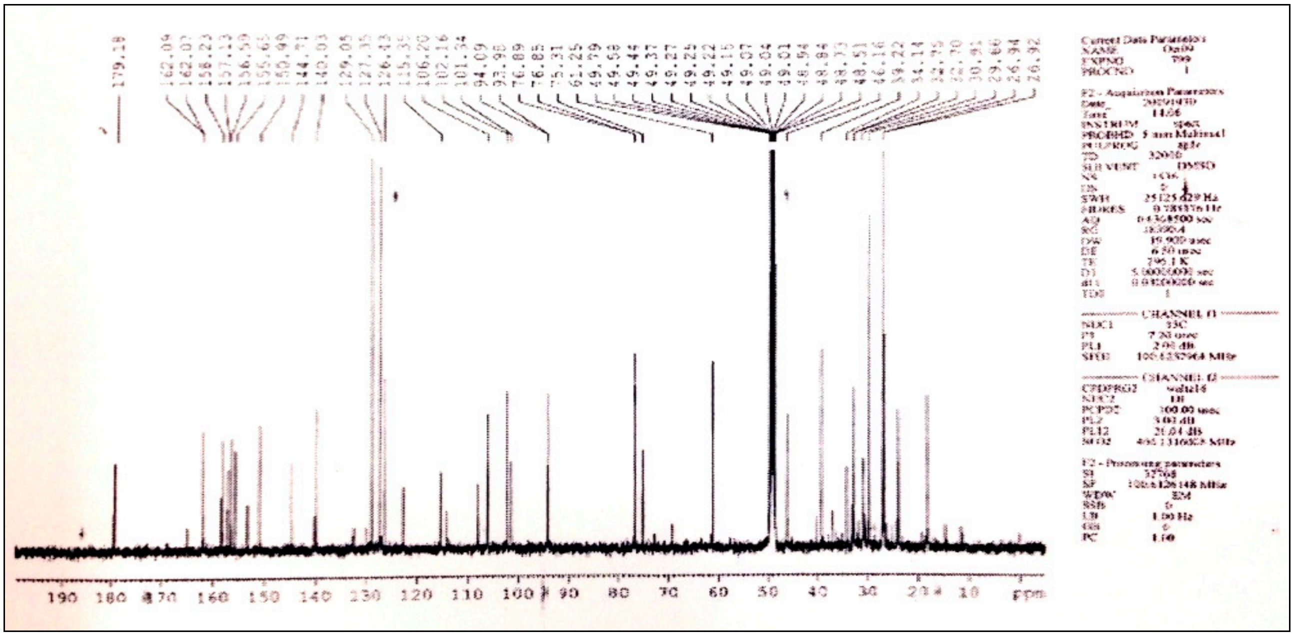

Table 4: NMR Spectral data of (I C)
NMR: 400MHz (Solvent: CD_3OD, δ, ppm), ppm: parts per million

Atom	^{13} C NMR (δ ppm)	^{1}H NMR (δ ppm)
1	162.09	-
2	106.20	-
3	164.97	-
4	94.09	6.62 (s, 1H)
5	102.16	6.28 (s, 1H)
6	162.07	-
7	144.71	-
8	140.03	-
9	179.86	-
10	101.34	-
11	158.23	-
12	115.35	-
13	157.13	-
14	18.23	2.37 (m, 2H)
15	46.16	2.65 (m, 2H)
16	61.25	-
17	26.92	1.24 - 1.43 (s, 3H)
18	26.94	1.24 - 1.43 (s, 3H)
19	29.66	3.07 (s, 2H)
20	30.91	1.84 (m, 2H)
21	76.89	-
22	32.70	1.24 - 1.43 (s, 3H)
23	32.75	1.24 - 1.43 (s, 3H)
-NH**CO**$CH_2CH_2CH_3$	179.18	-
-NHCO**CH_2**CH_2CH_3	39.22	1.92 (m, 2H)
-NHCOCH_2**CH_2**CH_3	18.23	1.24 - 1.43 (s, 2H)
-NHCOCH_2CH_2**CH_3**	14.28	1.24 - 1.43 (s, 3H)
OCH_3	61.25	3.55 (s, 3H)

5. Alkylated product of α-Mangostin (I D)

Fig 5a: Mass Spectrum of (I D)

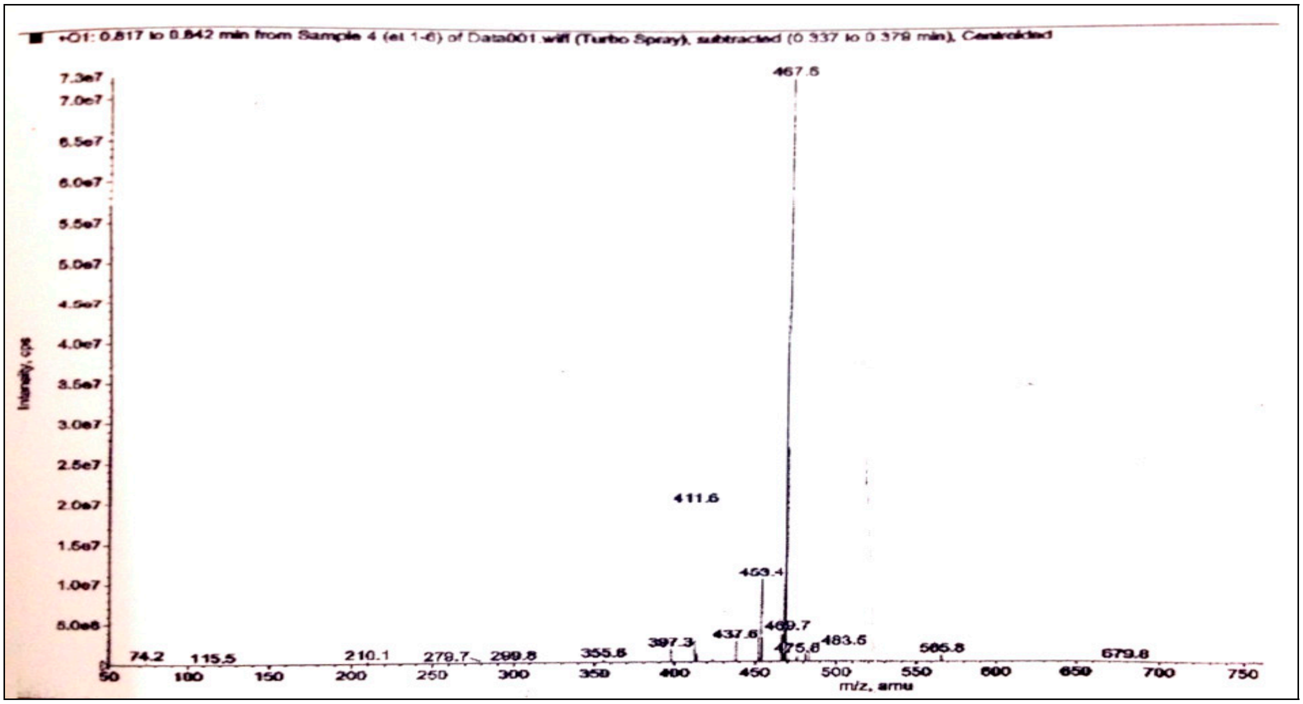

Fig 5b: ^{1}H NMR Spectrum of (I D)

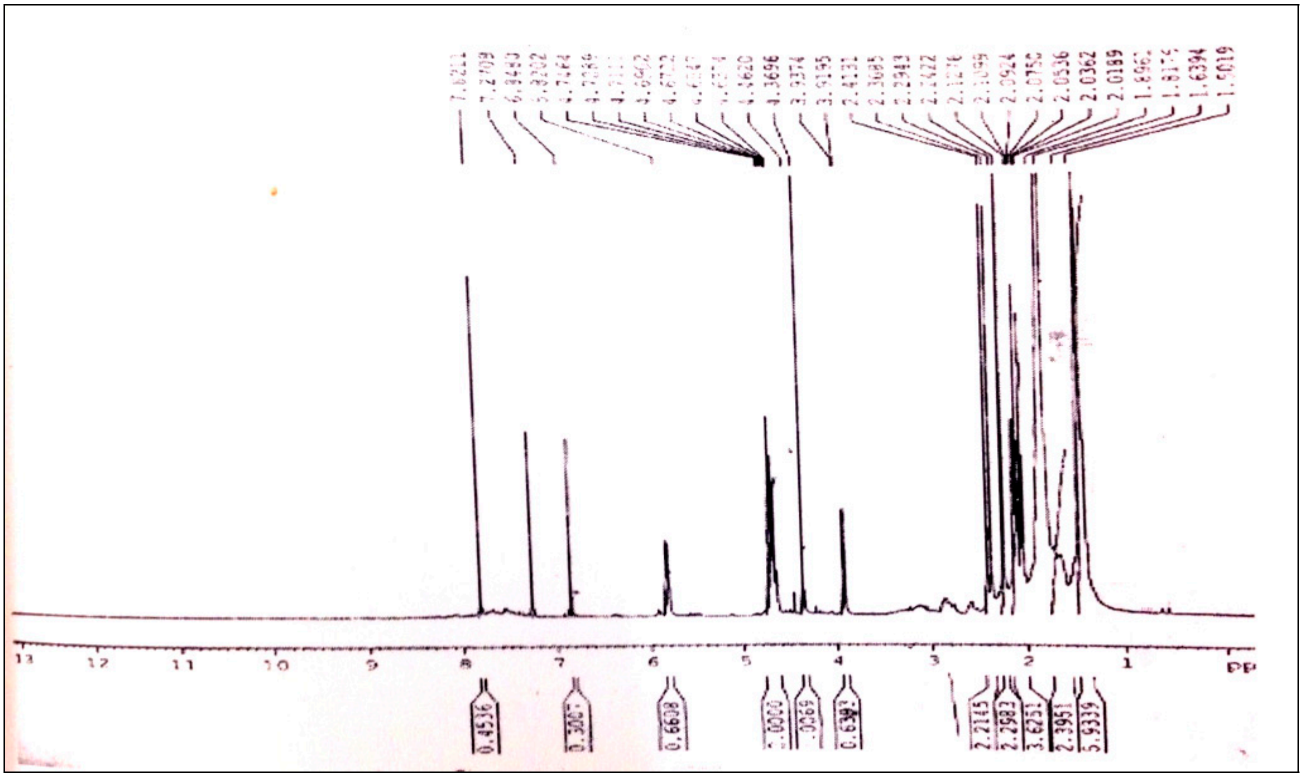

Fig 5c: 13 C NMR Spectrum of (I D)

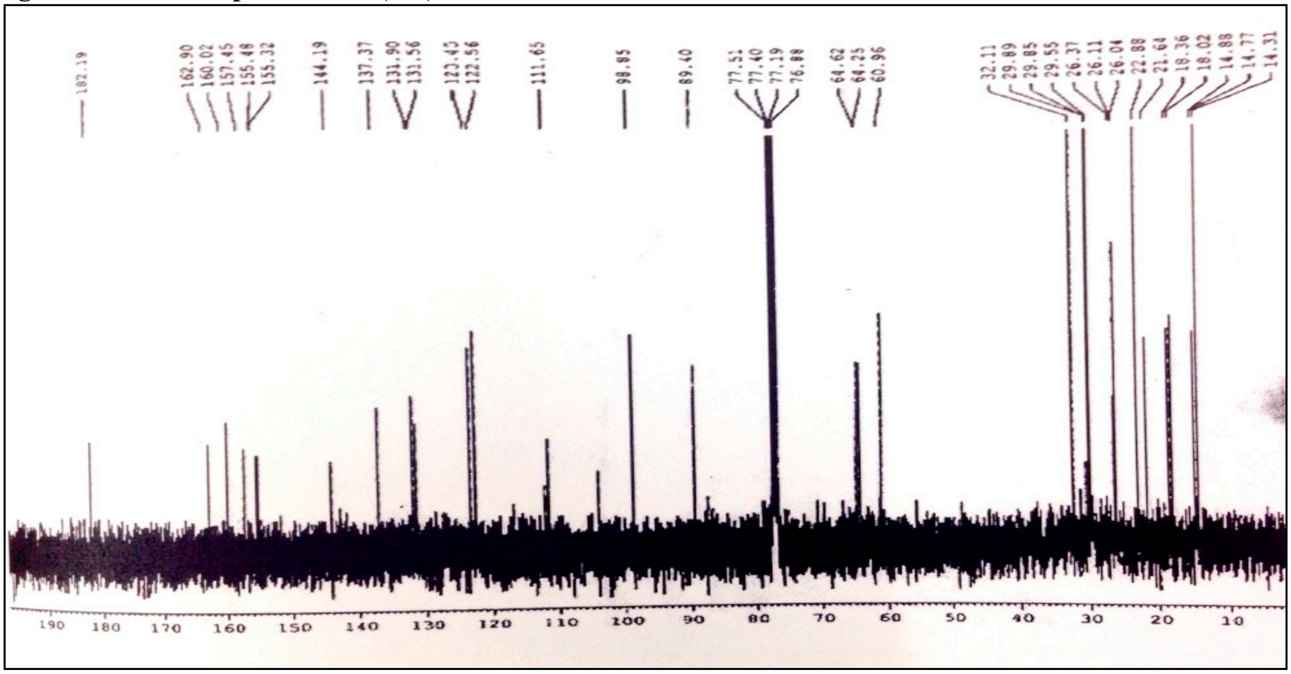

Table 5: NMR Spectral data of (I D)

NMR: 400MHz (Solvent: $CDCl_3$, δ, ppm), ppm: parts per million

Atom	13 C NMR (δ ppm)	^{1}H NMR (δ ppm)
1	160.02	-
2	111.65	-
3	162.90	-
4	89.40	6.84 (s, 1H)
5	103.82	7.82 (s, 1H)
6	155.32	-
7	144.19	-
8	137.37	-
9	182.19	-
10	98.85	-
11	157.45	-
12	111.80	-
13	155.48	-
14	26.04	3.91-3.93 (m,2H)
15	123.45	5.82 (s, 1H)
16	131.90	-
17	22.88	1.81-1.89 (s, 3H)
18	26.11	1.81-1.89 (s, 3H)
19	18.36	3.91-3.93 (s, 2H)
20	122.56	5.82 (m,1H)
21	131.56	-
22	26.37	1.81-1.89 (s, 3H)
23	22.88	1.81-1.89 (s, 3H)
24 & 24’	64.42 & 64.25	4.63-4.74 (m, 4H)
25 & 25’	14.31 – 14.88	1.39-1.50 (m, 6H)
OCH_3	60.96	3.93 (s, 3H)

6. Alkylated product of α-Mangostin (I E)

Fig 6a: Mass Spectrum of (I E)

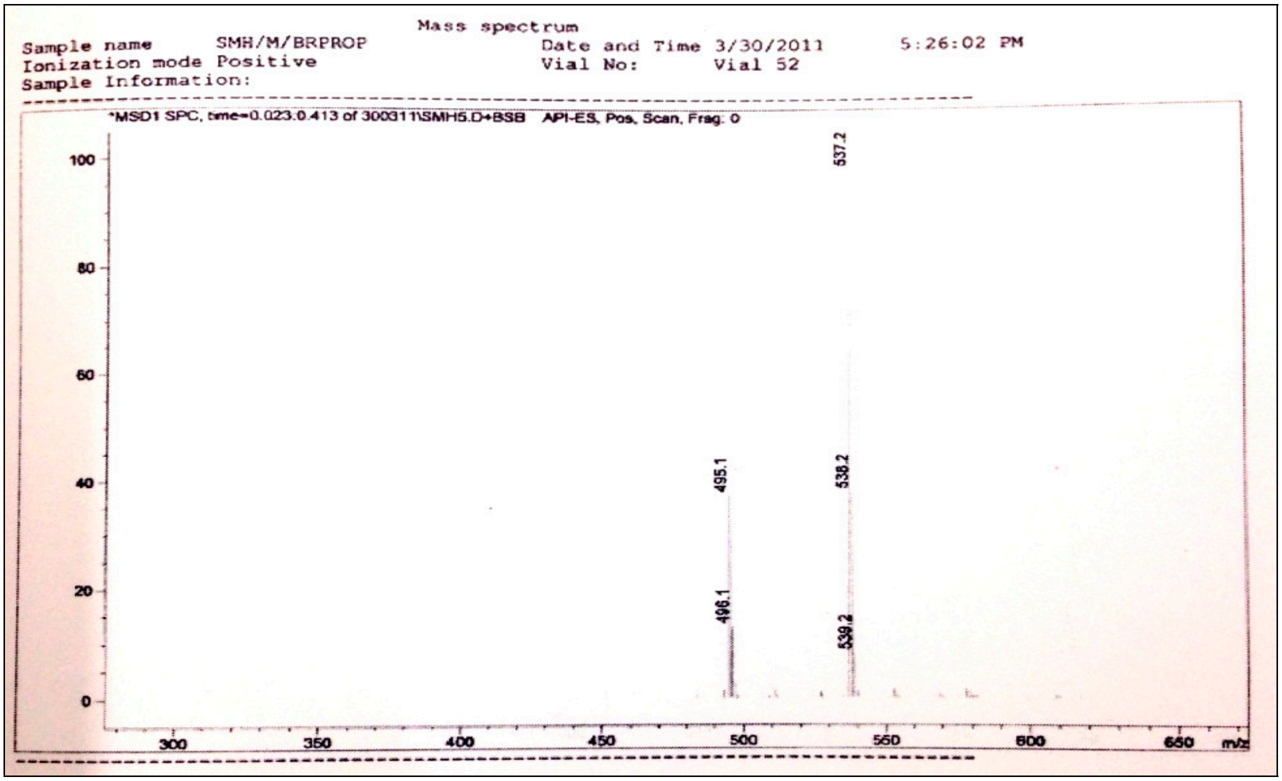

Fig 6b: ^{1}H NMR Spectrum of (I E)

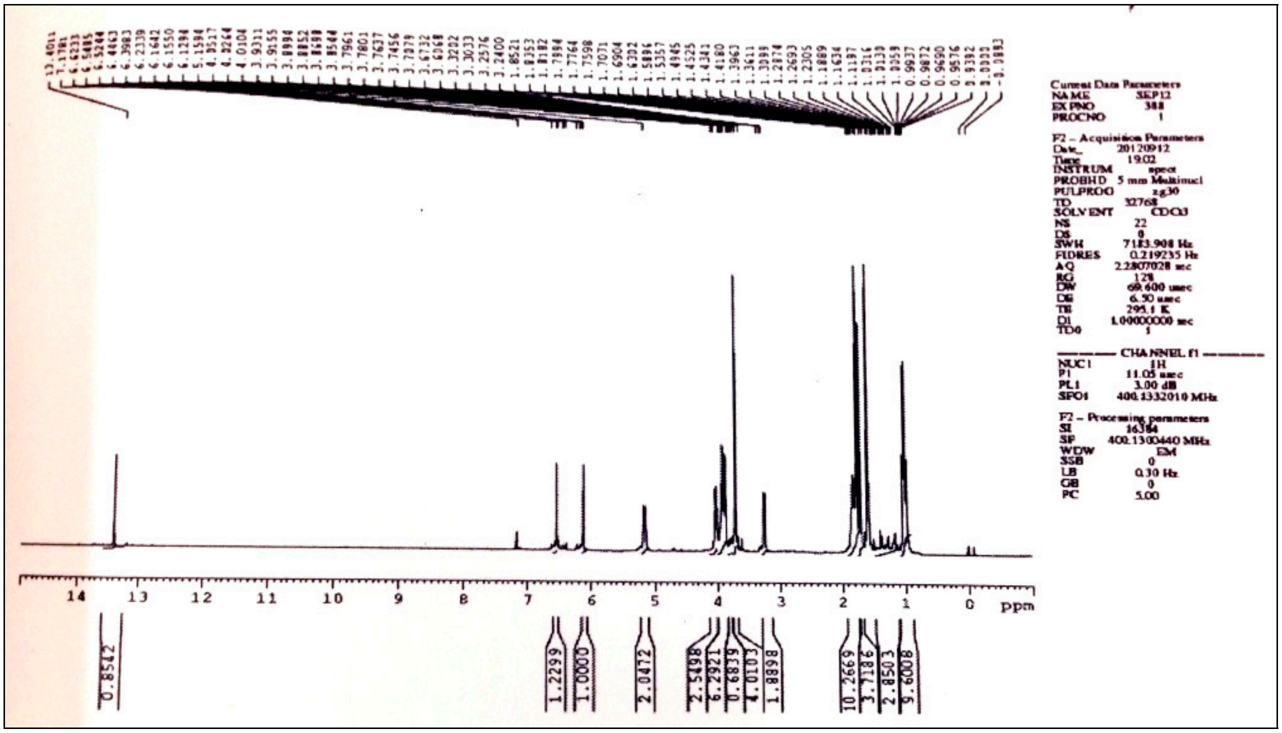

Fig 6c: ^{13} C NMR Spectrum of (I E)

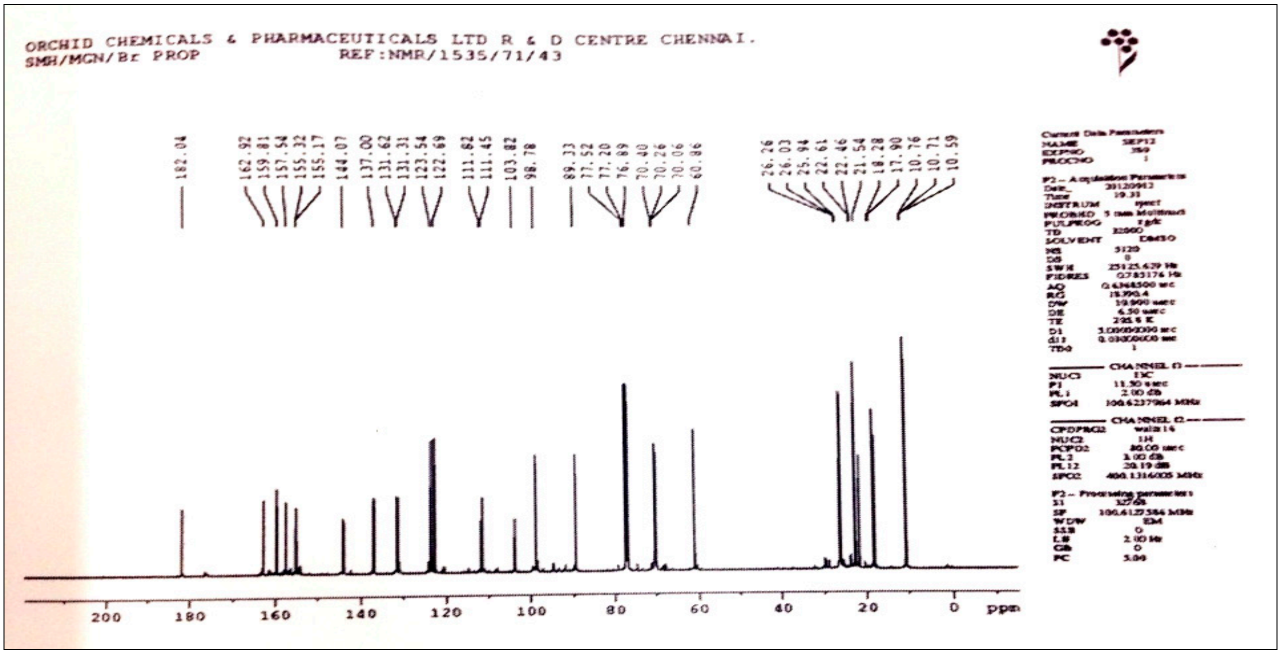

Table 6: NMR Spectral data of (I E)

NMR: 400MHz (Solvent: $CDCl_3$, δ, ppm), ppm: parts per million

Atom	^{13} C NMR (δ ppm)	^{1}H NMR (δ ppm)
1	159.81	-
2	111.45	-
3	162.92	-
4	89.33	6.12 (s, 1H)
5	103.82	6.12 (s, 1H)
6	155.17	-
7	144.07	-
8	137.08	-
9	182.04	-
10	98.78	-
11	157.54	-
12	111.82	-
13	155.32	-
14	25.94	3.24-3.32 (m, 2H)
15	123.54	5.15 (m, 1H)
16	131.62	-
17	10.78	1.70-1.85 (s, 3H)
18	25.94	1.70-1.85 (s, 3H)
19	21.54	3.24-3.32 (m, 2H)
20	122.69	5.15 (m, 1H)
21	131.31	-
22	25.94	1.70-1.85 (s, 3H)
23	18.28	1.70-1.85 (s, 3H)
24, 24' & 24"	70.06-70.40	3.85-3.93 (m, 6H)
25, 25 & 25"	21.54-22.61	1.58 (m, 6H)
26, 26' & 26"	10.59-10.76	0.93-1.03 (m, 9H)
OCH_3	60.86	3.70 (s, 3H)

7. Alkylated product of α-Mangostin (I F)

Fig 7a: Mass Spectrum of (I F)

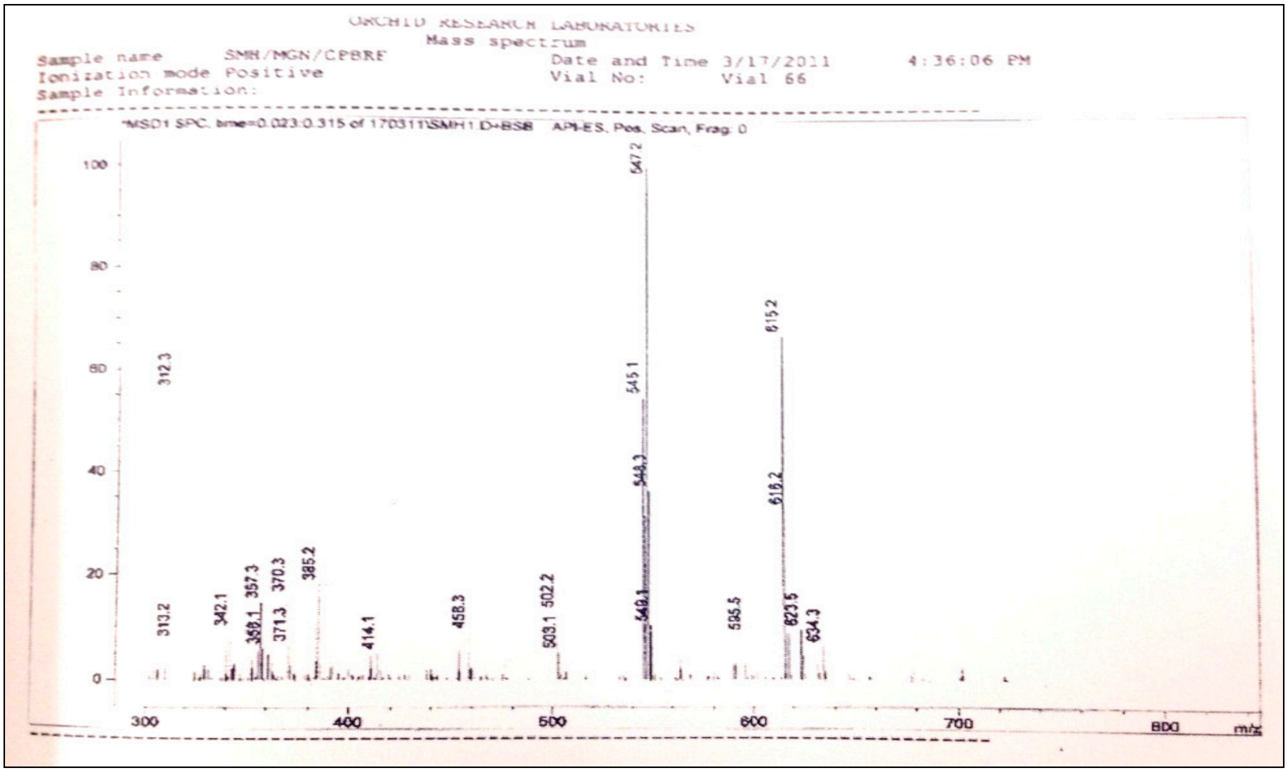

Fig 7b: ^{1}H NMR Spectrum of (I F)

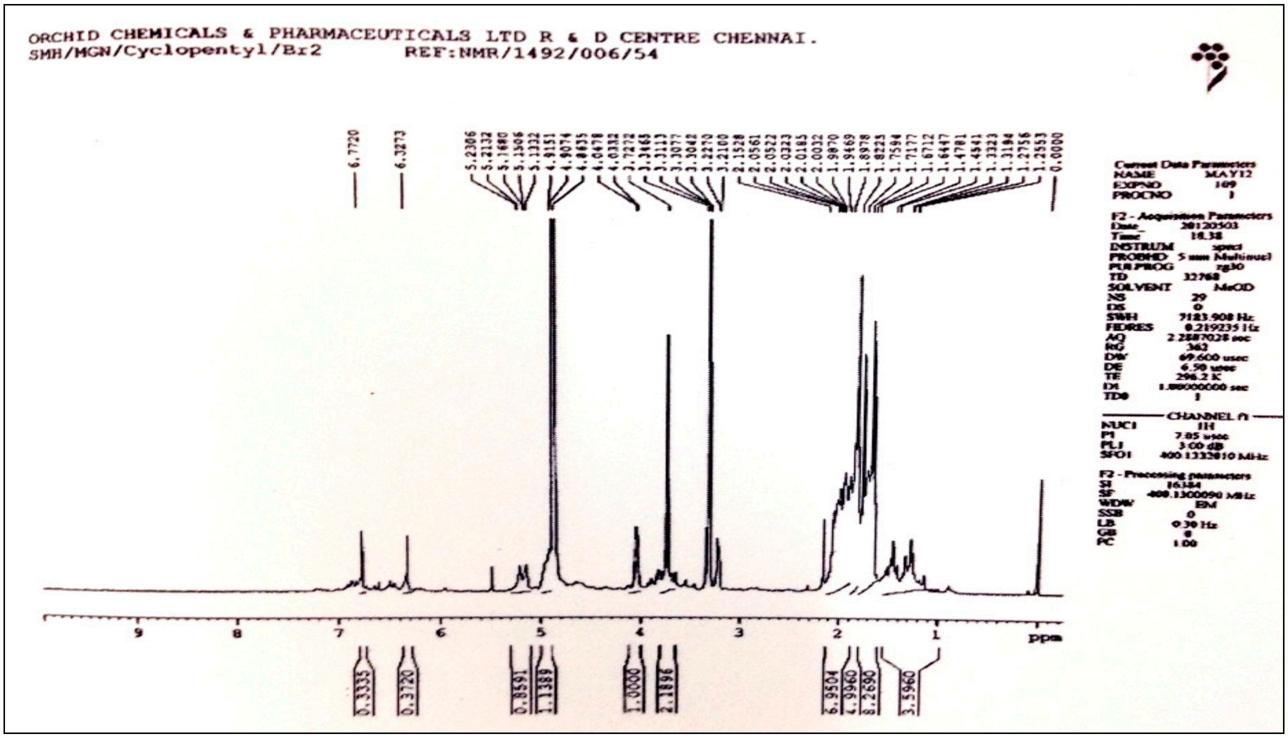

Fig 7c: [13] C NMR Spectrum of (I F)

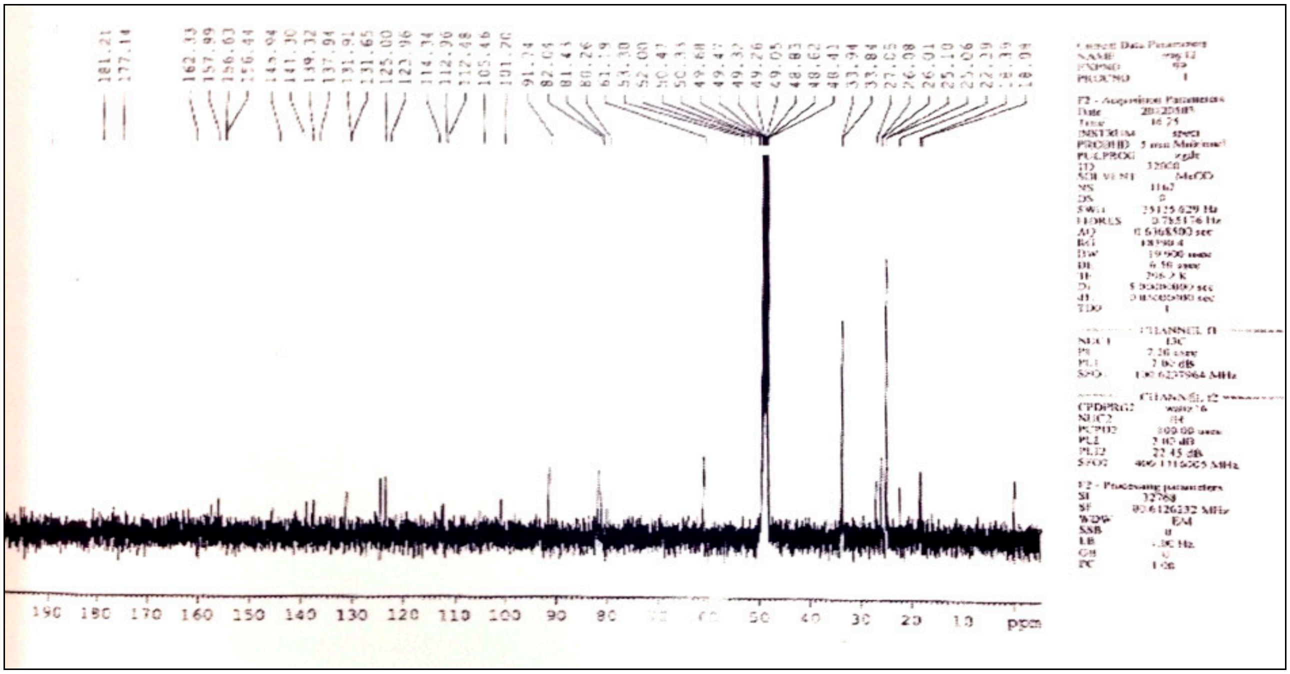

Table 7: NMR Spectral data of (I F)

NMR: 400MHz (Solvent: CD_3OD, δ, ppm), ppm: parts per million

Atom	^{13} C NMR (δ ppm)	^{1}H NMR (δ ppm)
1	159.63	-
2	112.48	-
3	162.33	-
4	91.74	6.32 (s, 1H)
5	105.46	6.77 (s, 1H)
6	156.44	-
7	145.94	-
8	137.94	-
9	181.21	-
10	101.20	-
11	157.99	-
12	112.96	-
13	156.63	-
14	26.08	3.21-3.22 (m, 2H)
15	123.96	5.28 (m, 1H)
16	131.65	-
17	18.08	1.82 (s, 3H)
18	25.10	1.82 (s, 3H)
19	22.39	3.21-3.22 (m, 2H)
20	123.96	5.28 (m, 1H)
21	131.91	-
22	27.05	1.82 (s, 3H)
23	18.39	1.82 (s, 3H)
O-**CH-**CP	80.26, 81.43, 82.04	3.72 (m, 3H)
O-Cypentyl	33.84	1.71-1.75 (m, 2H)
OCH_3	61.19	3.30 (s, 3H)

8. Alkylated product of α-Mangostin (I G)

Fig 8a: Mass Spectrum of (I G)

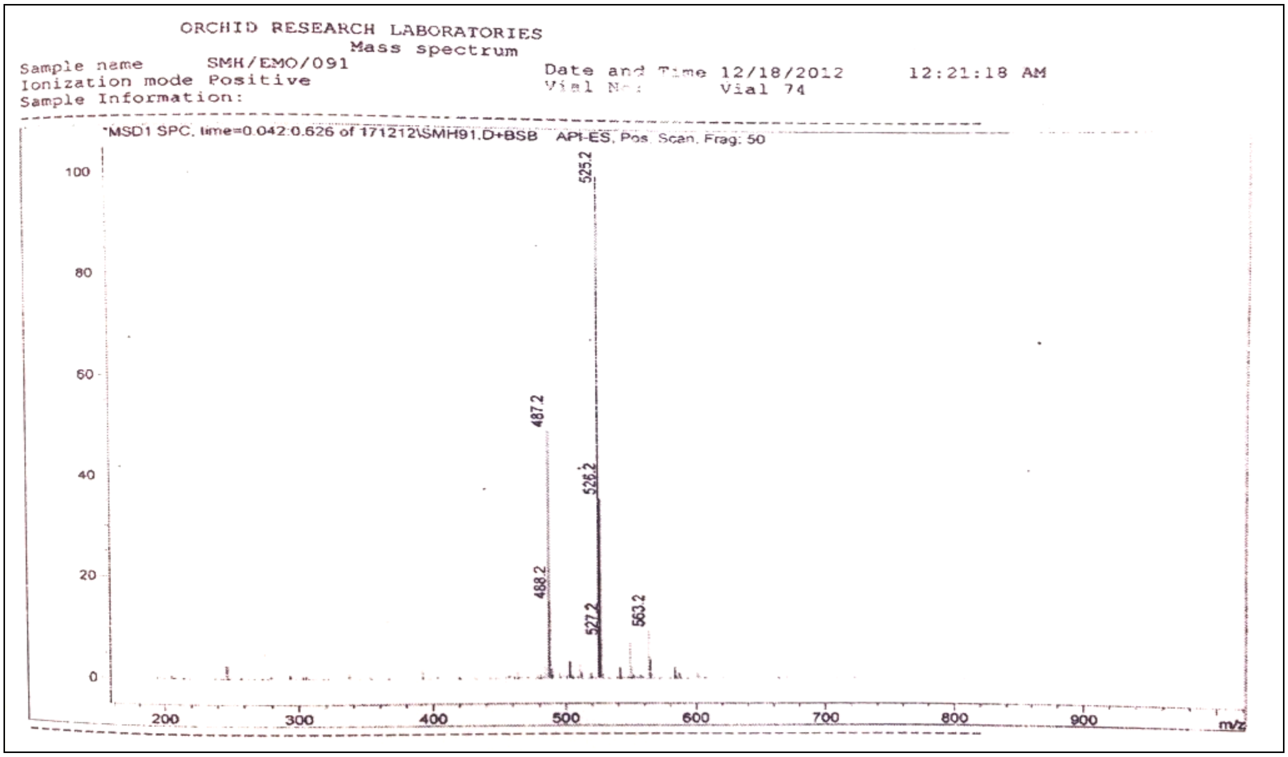

Fig 8b: ^{1}H NMR Spectrum of (I G)

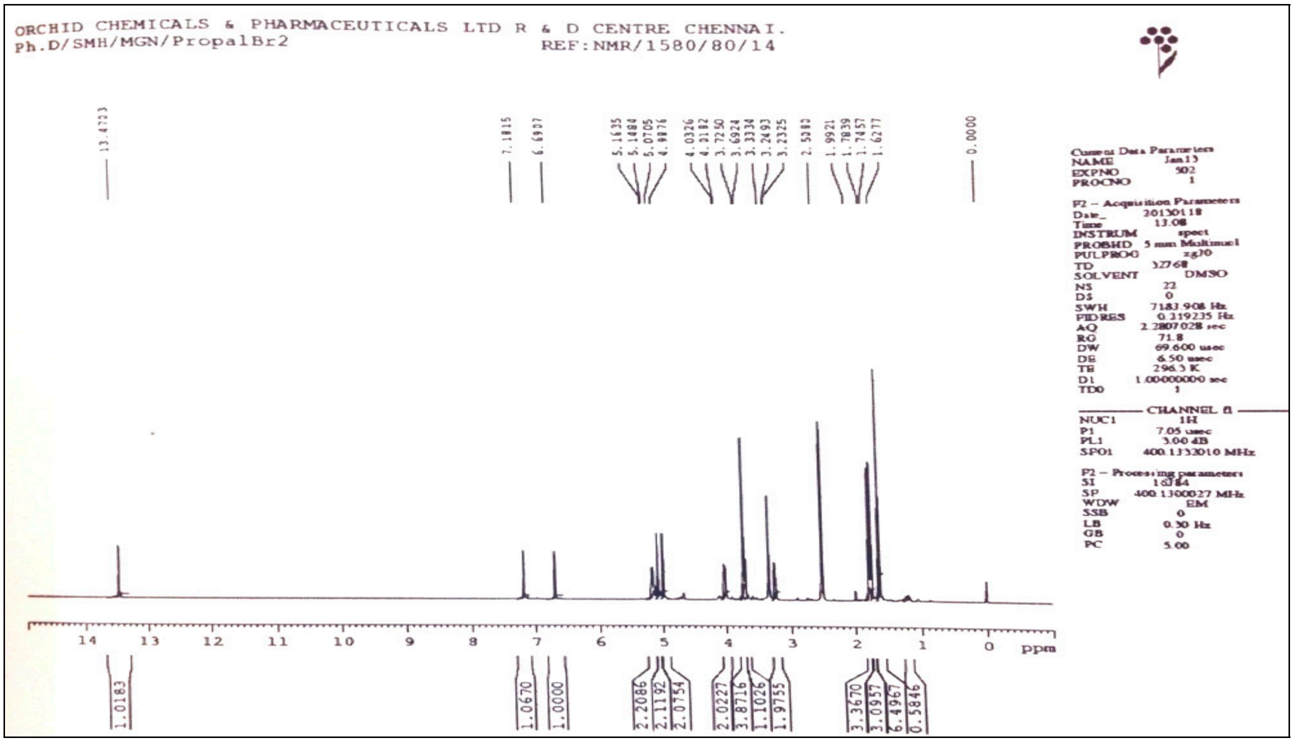

Fig 8c: ^{13} C NMR Spectrum of (I G)

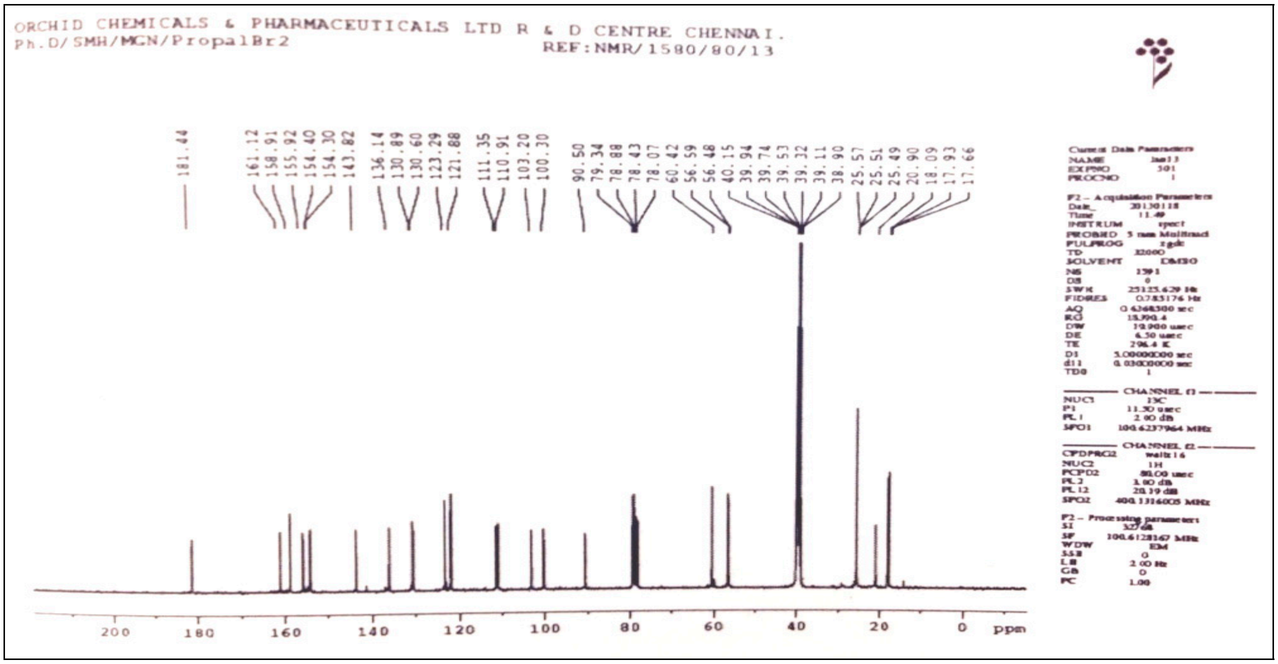

Table 8: NMR Spectral data of (I G)
NMR: 400MHz (Solvent: DMSO-d$_6$, δ, ppm), ppm: parts per million

Atom	^{13} C NMR (δ ppm)	^{1}H NMR (δ ppm)
1	158.91	-
2	110.91	-
3	161.12	-
4	100.30	6.69 (s, 1H)
5	103.20	7.18 (s, 1H)
6	154.30	-
7	143.82	-
8	130.89	-
9	181.44	-
10	111.35	-
11	155.92	-
12	130.60	-
13	154.40	-
14	20.90	3.23-3.33 (m, 2H)
15	123.29	5.14 (m, 1H)
16	136.14	-
17	25.57	1.62-1.78 (s, 3H)
18	17.93	1.62-1.78 (s, 3H)
19	18.09	3.23-3.33 (m, 2H)
20	121.88	5.14 (m, 1H)
21	136.14	-
22	25.51	1.62-1.78 (s, 3H)
23	17.66	1.62-1.78 (s, 3H)
24	60.42	4.01-5.08 (m, 6H)
25	79.34	-
26	78.43	1.99(m, 3H)
OCH_3	56.48	3.72 (s, 3H)

9. Alkylated product of α-Mangostin (I H)

Fig 9a: Mass Spectrum of (I H)

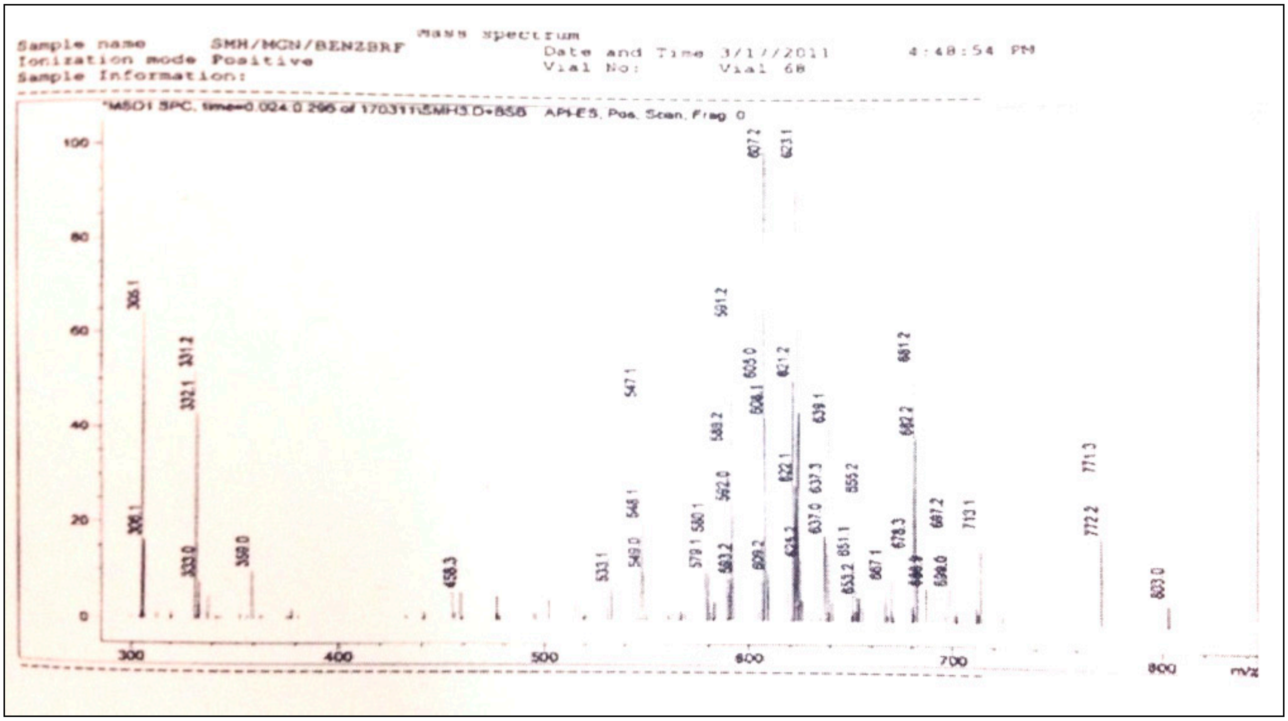

Fig 9b: ^{1}H NMR Spectrum of (I H)

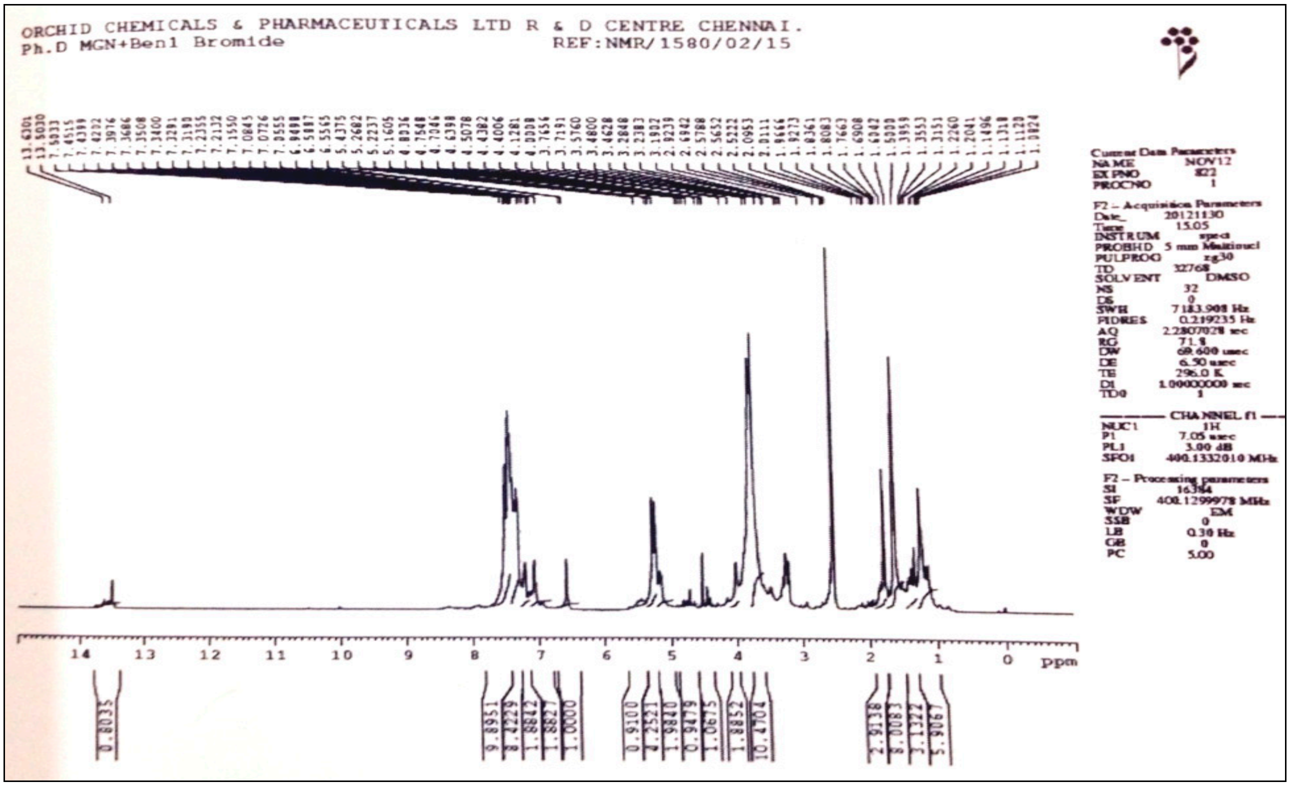

Fig 9c: ^{13} C NMR Spectrum of (I H)

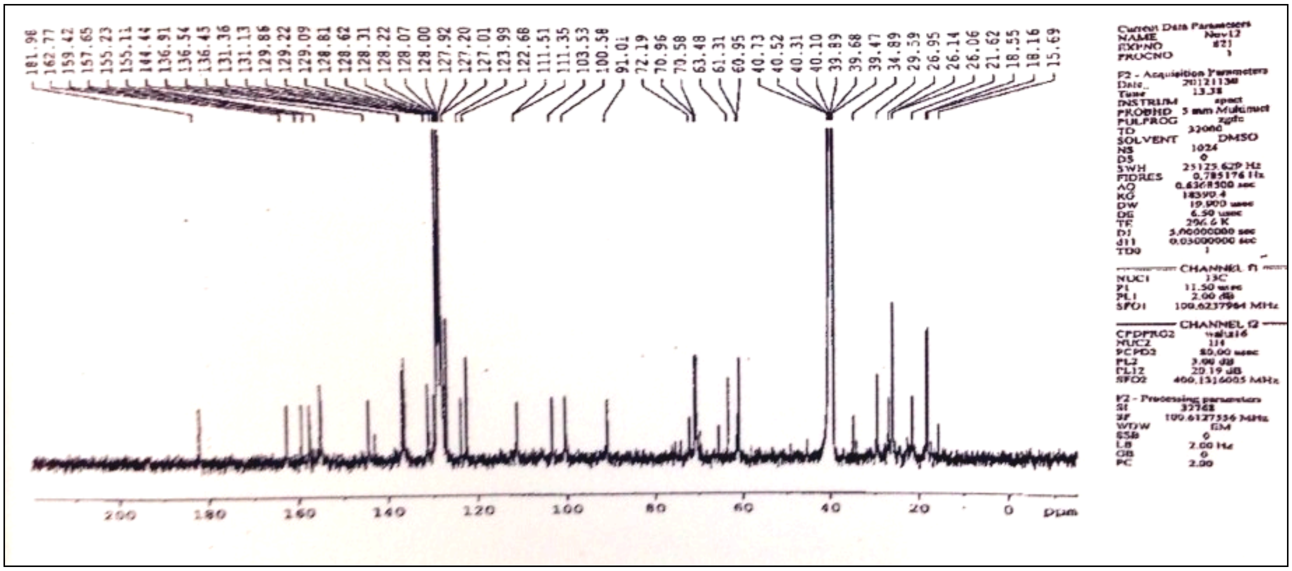

Table 9: NMR Spectral data of (I H)
NMR: 400MHz (Solvent: DMSO-d$_6$, δ, ppm), ppm: parts per million

Atom	^{13} C NMR (δ ppm)	^{1}H NMR (δ ppm)
1	159.42	-
2	100.58	-
3	162.77	-
4	91.01	6.58(s, 1H)
5	100.58	7.08 (s, 1H)
6	155.11	-
7	144.44	-
8	123.99	-
9	181.80	-
10	103.53	-
11	157.65	-
12	122.68	-
13	155.23	-
14	21.62	3.23-3.38(m, 2H)
15	111.51	5.16(m, 1H)
16	131.36	-
17	26.14	1.50-1.80 (s, 3H)
18	18.55	1.50-1.80 (s, 3H)
19	15.69	3.23-3.28(m, 2H)
20	111.35	5.16(m, 1H)
21	131.13	-
22	26.06	1.50-1.80 (s, 3H)
23	18.16	1.50-1.80 (s, 3H)
24,24',24"	70.58-72.19	5.16-5.22 (m, 6H)
25,25',25"	136.45-136.91	-
26,26',26"	127.01-129.86	7.31-7.50(m, 3H)
26a,26a',26a"	127.01-129.86	7.31-7.50(m, 3H
27,27',27"	127.01-129.86	7.31-7.50(m, 3H
27a,27a',27a"	127.01-129.86	7.31-7.50(m, 3H
28,28',28"	127.01-129.86	7.31-7.50(m, 3H
OCH_3	60.95	3.78(s, 3H)

10. Alkylated product of α-Mangostin (I I)

Fig 10a: Mass Spectrum of (I I)

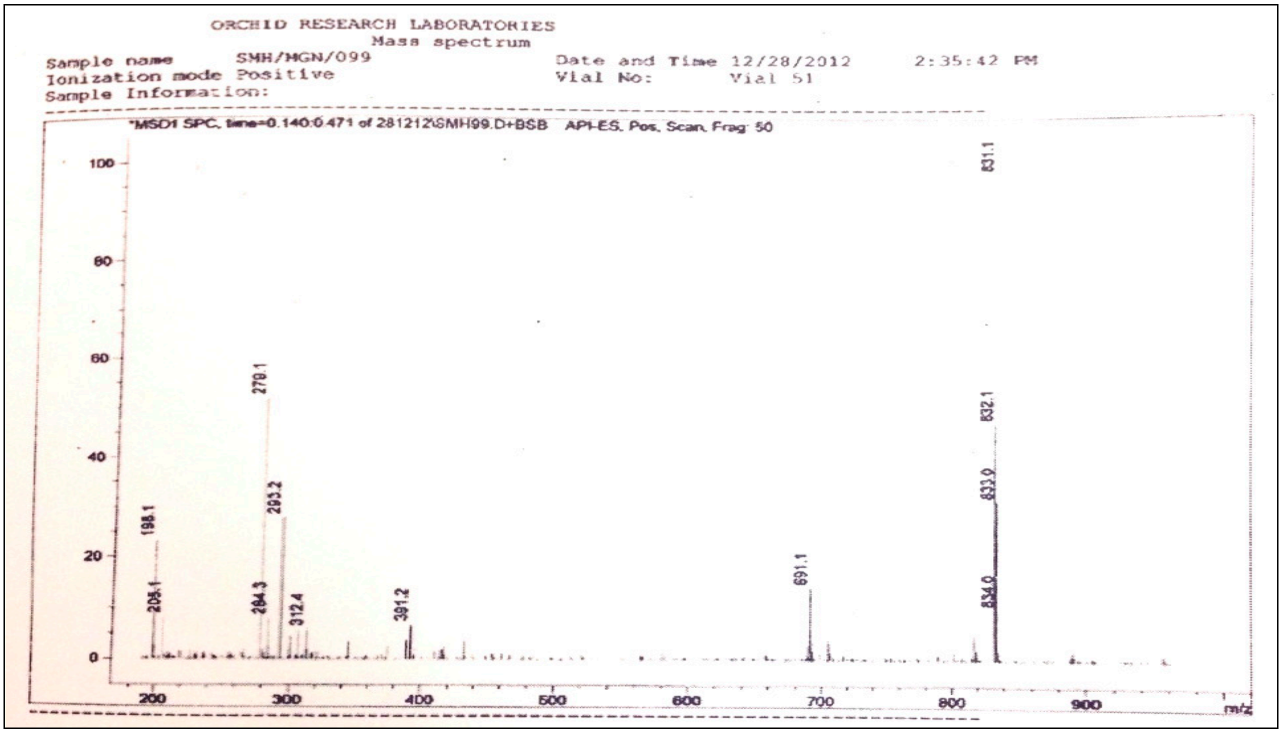

Fig 10b: ^{1}H NMR Spectrum of (I I)

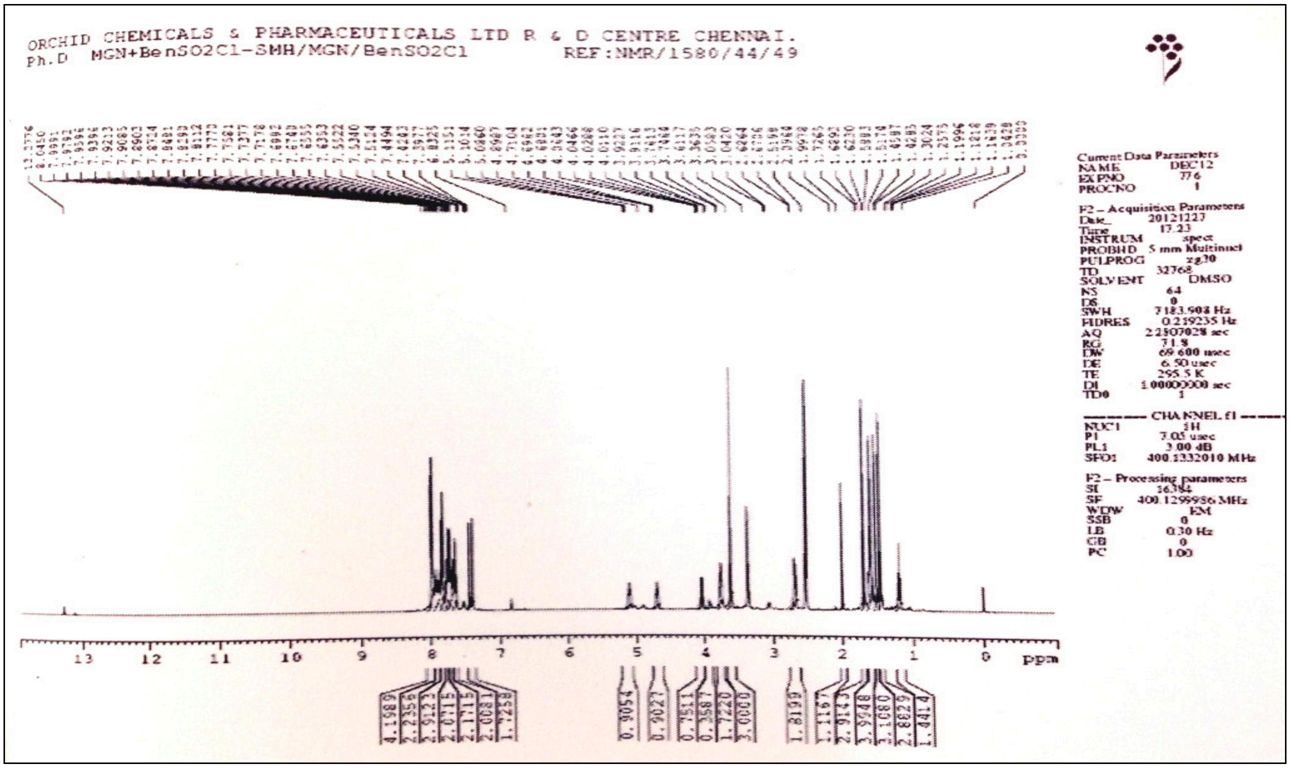

Fig 10c: ^{13} C NMR Spectrum of (I I)

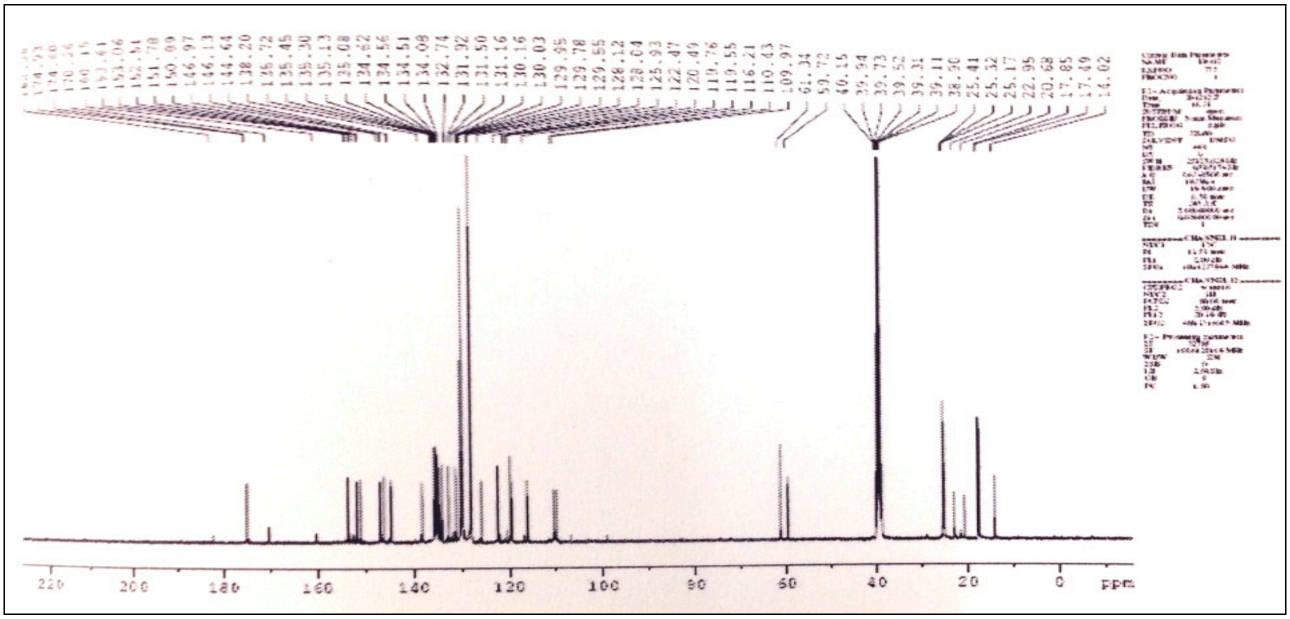

Table 10: NMR Spectral data of (I I)

NMR: 400MHz (Solvent: DMSO-d$_6$, δ, ppm), ppm: parts per million

Atom	^{13} C NMR (δ ppm)	^{1}H NMR (δ ppm)
1	151.78	-
2	116.21	-
3	153.61	-
4	109.97	7.42 (s, 1H)
5	110.43	7.38 (s, 1H)
6	146.13	-
7	144.64	-
8	125.93	-
9	174.93	-
10	119.55	-
11	150.89	-
12	122.47	-
13	146.97	-
14	20.68	3.36(m, 2H)
15	120.49	5.11(m, 1H)
16	138.20	-
17	25.41	1.04-1.98(s, 3H)
18	17.49	1.04-1.98(s, 3H)
19	14.02	3.36(m, 2H)
20	119.76	4.88(m, 1H)
21	135.72	-
22	25.17	1.04-1.98(s, 3H)
23	17.85	1.04-1.98(s, 3H)
24	128.04-135.45	-
25	128.04-135.45	7.53-8.04(m, 3H)
26	128.04-135.45	7.53-8.04(m, 3H)
26a	128.04-135.45	7.53-8.04(m, 3H)
27	128.04-135.45	7.53-8.04(m, 3H)
27a	128.04-135.45	7.53-8.04(m, 3H)
OCH_3	61.34	3.61(s, 3H)

11. Alkylated product of α-Mangostin (I J)

Fig 11a: Mass Spectrum of (I J)

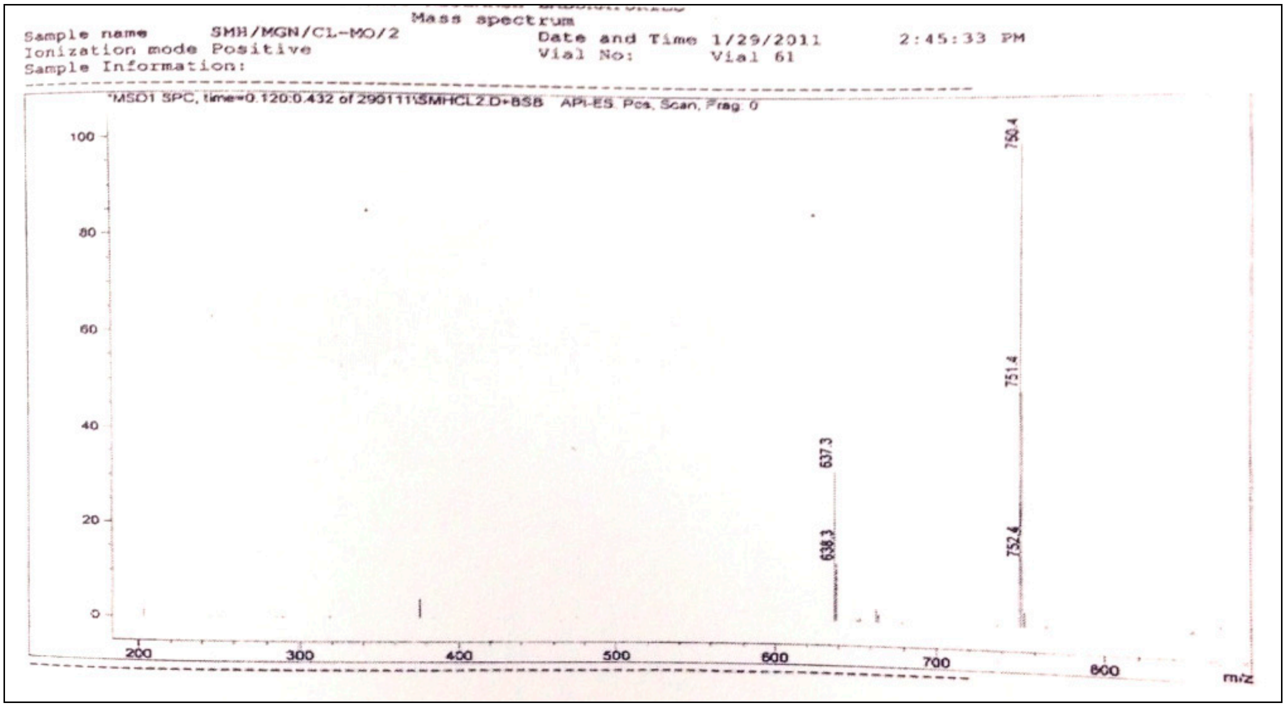

Fig 11b: ^{1}H NMR Spectrum of (I J)

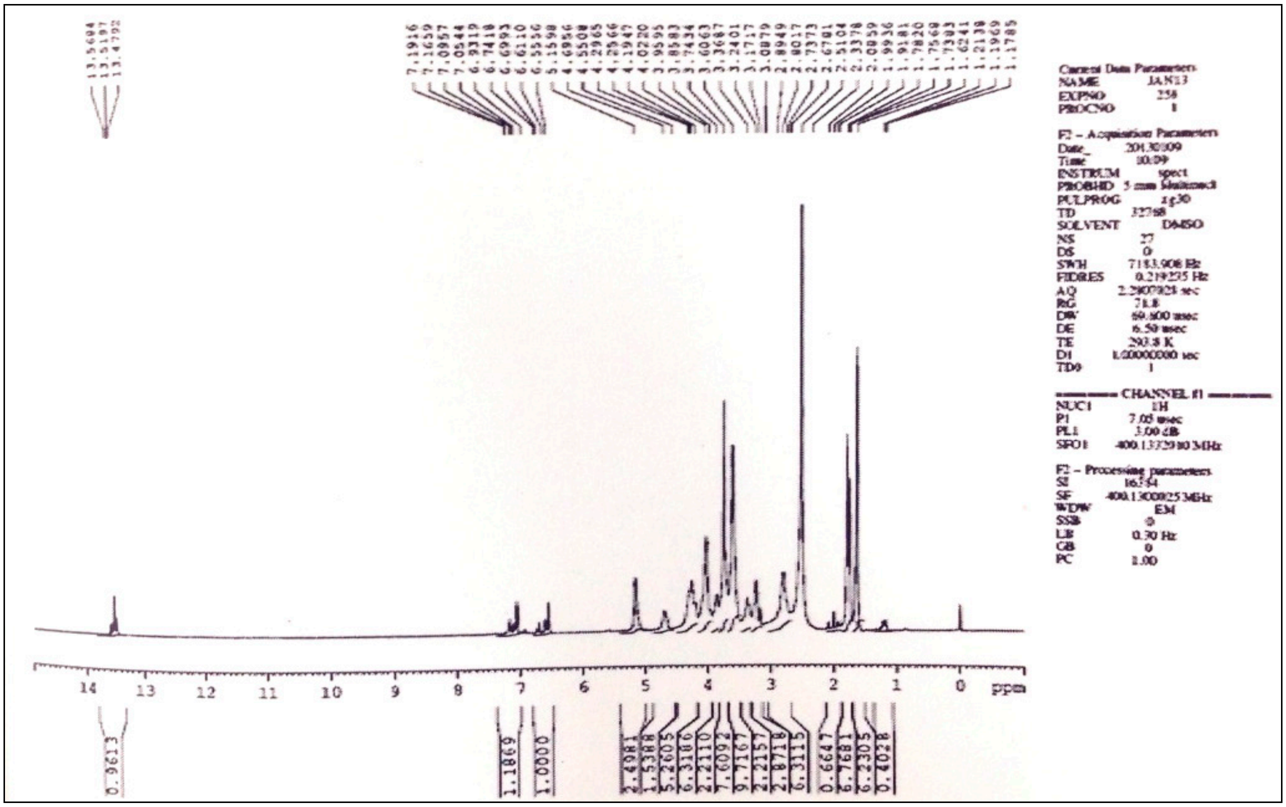

Fig 11c: ^{13}C NMR Spectrum of (I J)

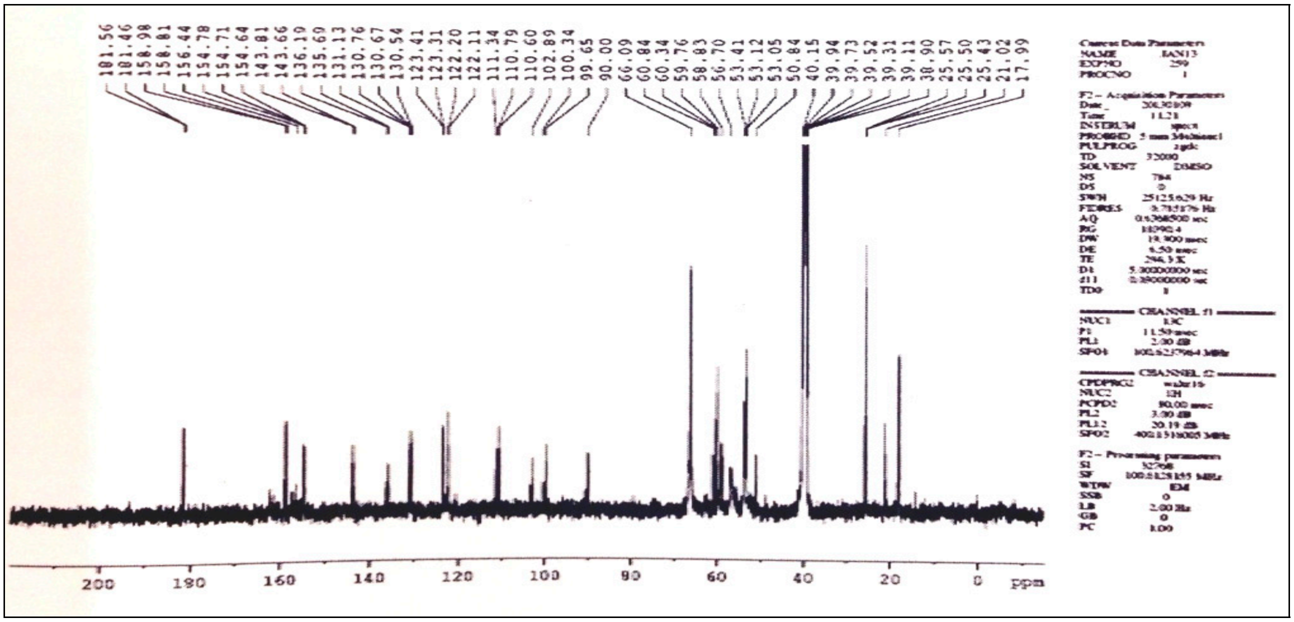

Table 11: NMR Spectral data of (I J)

NMR: 400MHz (Solvent: DMSO-d_6, δ, ppm), ppm: parts per million

Atom	^{13}C NMR (δ ppm)	^{1}H NMR (δ ppm)
1	158.81	-
2	100.34	-
3	158.98	-
4	90.00	6.55(s, 1H)
5	99.65	7.08(s, 1H)
6	154.64	-
7	143.81	-
8	123.41	-
9	181.56	-
10	102.89	-
11	156.44	-
12	122.20	-
13	154.71	-
14	21.02	3.17-3.24(m, 2H)
15	111.34	5.15(m, 1H)
16	136.19	-
17	25.57	1.62-1.78(s, 3H)
18	25.50	1.62-1.78(s, 3H)
19	17.99	3.17-3.24(m, 2H)
20	110.80	5.15(m, 1H)
21	135.69	-
22	25.57	1.62-1.78(s, 3H)
23	25.43	1.62-1.78(s, 3H)
24	66.09	4.18(m, 6H)
25	53.12	3.60(m, 6H)
26	59.76	2.67-3.08(m, 6H)
26a	58.83	2.67-3.08(m, 6H)
27	60.84	3.74-4.02(m, 6H)
27a	60.34	3.74-4.02(m, 6H)
**OCH_3**	56.70	3.74(s, 3H)

12. Acylated product of α-Mangostin (I K)

Fig 12a: Mass Spectrum of (I K)

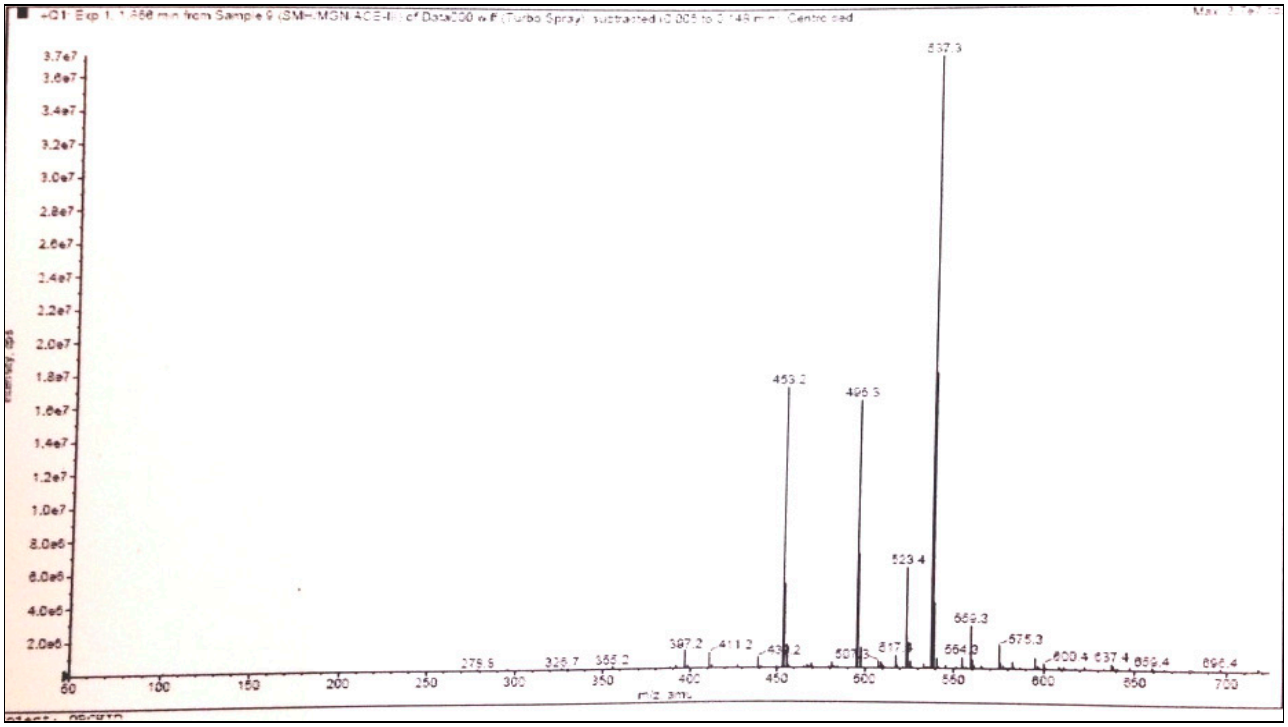

Fig 12b: ^{1}H NMR Spectrum of (I K)

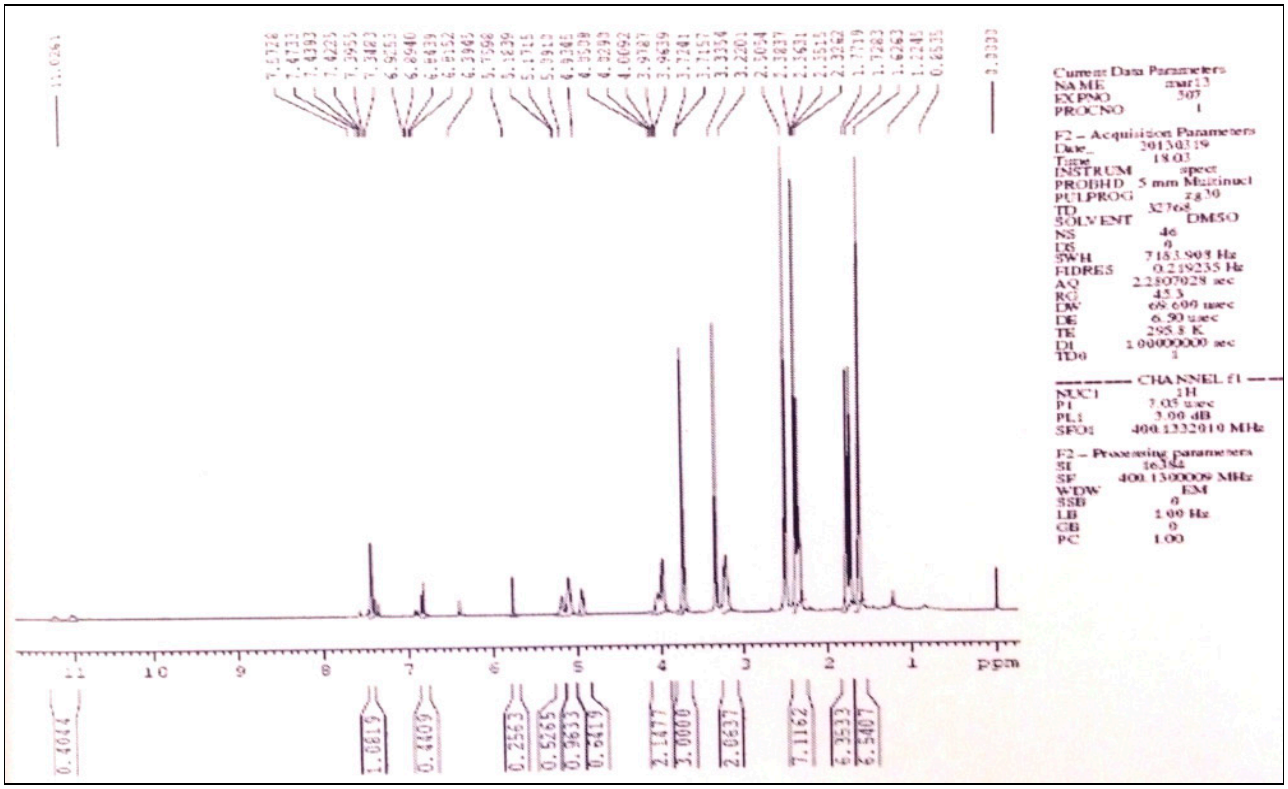

Fig 12c: ^{13} C NMR Spectrum of (I K)

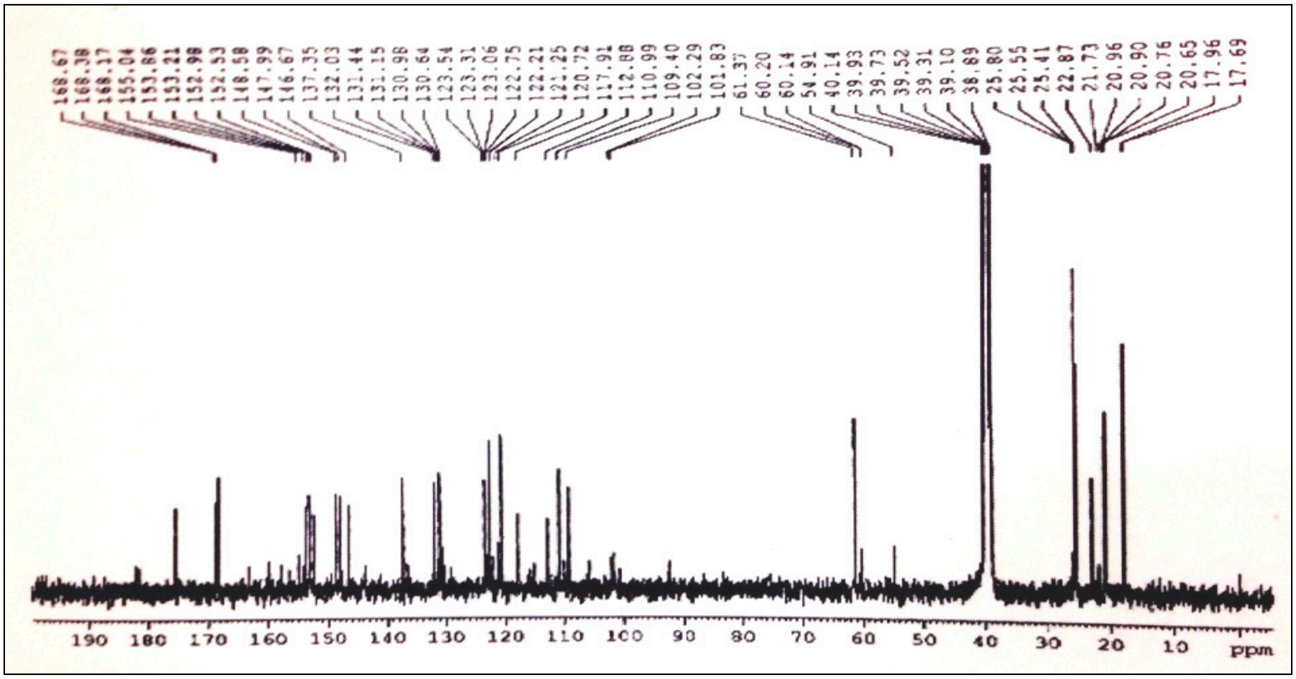

Table 12: NMR Spectral data of (I K)

NMR: 400MHz (Solvent: DMSO-d_6, δ, ppm), ppm: parts per million

Atom	^{13} C NMR (δ ppm)	^{1}H NMR (δ ppm)
1	155.04	-
2	112.88	-
3	153.86	-
4	109.40	6.89(s, 1H)
5	110.99	7.48(s, 1H)
6	152.53	-
7	148.58	-
8	123.31	-
9	175.47	-
10	117.91	-
11	153.21	-
12	122.75	-
13	152.98	-
14	22.87	3.22-3.33(m, 2H)
15	121.25	5.18(m, 1H)
16	137.35	-
17	25.41-25.80	1.62-1.78(s, 3H)
18	20.65-20.96	1.62-1.78(s, 3H)
19	21.73	3.22-3.33(m, 2H)
20	120.72	5.18(m, 1H)
21	132.03	-
22	25.41-25.80	1.62-1.78(s, 3H)
23	20.65-20.96	1.62-1.78(s, 3H)
24	168.67,168.38 & 168.17	-
25	17.69-17.96	2.35-2.38(m, 6H)
OCH_3	61.37	3.72

13. Reduced product of α-Mangostin (I L)

Fig 13a: Mass Spectrum of (I L)

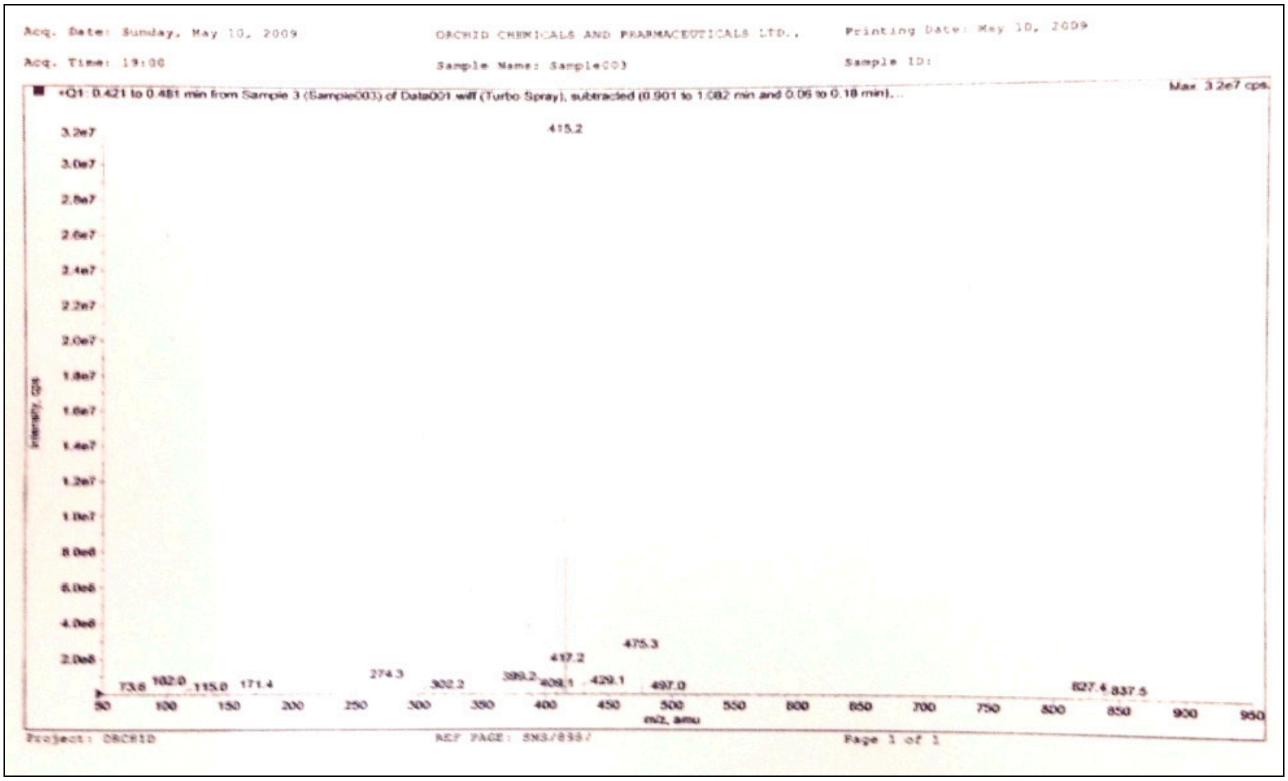

Fig 13b: ^{1}H NMR Spectrum of (I L)

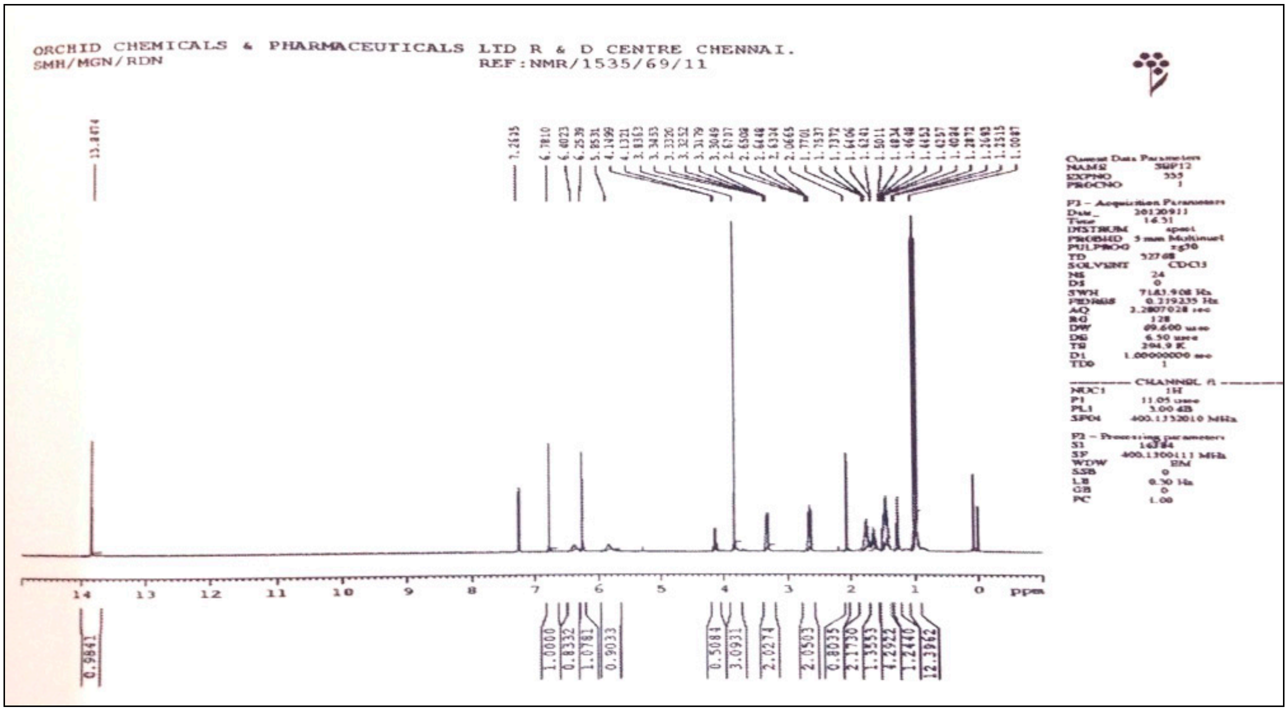

Fig 13c: ^{13} C NMR Spectrum of (I L)

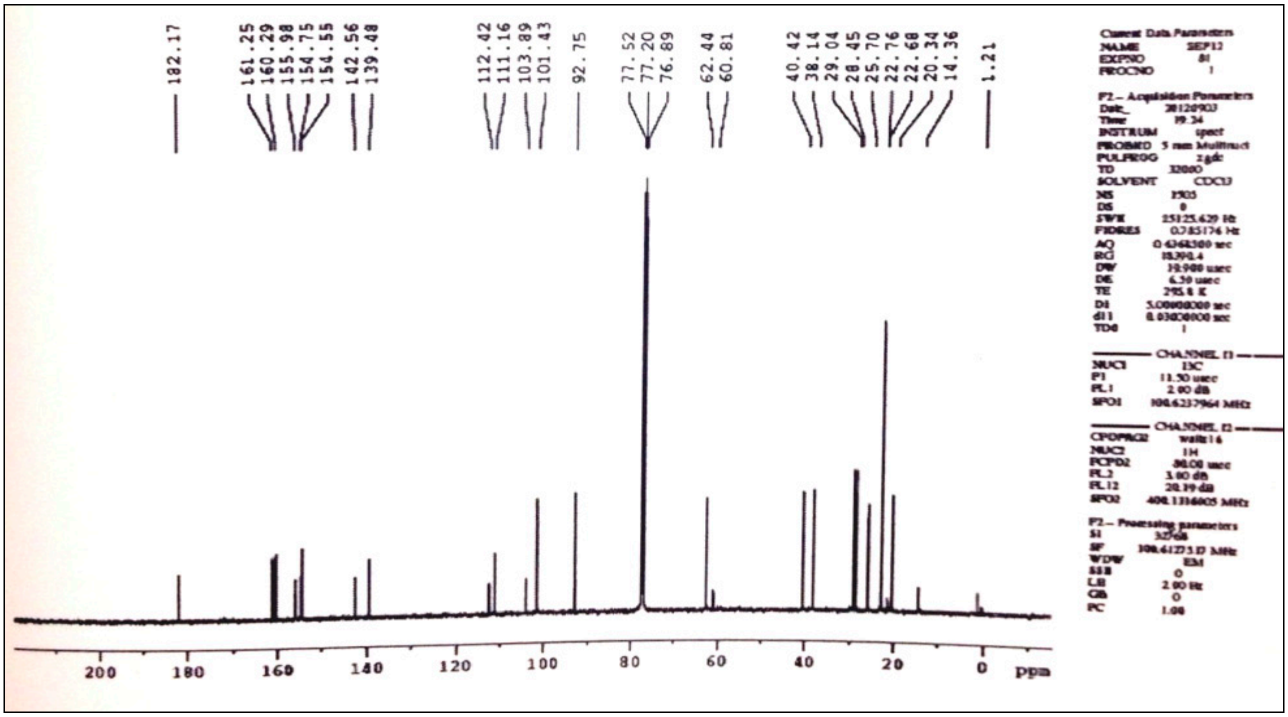

Table 13: NMR Spectral data of (I L)
NMR: 400MHz (Solvent: $CDCl_3$, δ, ppm), ppm: parts per million

Atom	^{13} C NMR (δ ppm)	^{1}H NMR (δ ppm)
1	161.25	-
2	111.16	-
3	160.29	-
4	92.75	6.78(s, 1H)
5	103.89	6.25(s, 1H)
6	154.55	-
7	142.56	-
8	139.48	-
9	182.17	-
10	101.43	-
11	155.98	-
12	112.42	-
13	154.75	-
14	20.34	2.65(t, 2H, J = 2.40Hz)
15	38.14	1.40-1.50(m, 2H)
16	28.45	1.26(m, 1H)
17	22.68	0.96(s, 3H)
18	22.68	0.98(s, 3H)
19	14.36	3.32(t, 2H, J = 2.72Hz)
20	40.42	1.40-1.50(m, 2H)
21	29.04	1.64(m, 1H)
22	22.76	0.99(s, 3H)
23	22.76	1.00(s, 3H)
OCH_3	62.44	3.83(s, 3H)
