# Supplementary material for: Anti-Bacterial and Anti-Fungal Activity of Xanthones Obtained via Semi-Synthetic Modification of α-Mangostin from Garcinia mangostana
Source: Molecules. 2017 Feb 12;22(2):275. doi: 10.3390/molecules22020275 (PMC6155947; doi:10.3390/molecules22020275)
Supplement: Supplementary file 2 [file molecules-22-00275-s002.pdf]

Supplementary Materials: Anti-Bacterial and Anti-Fungal Activity of Xanthones Obtained via Semi-Synthetic Modification of α-Mangostin from *Garcinia mangostana*

Srinivasan Narasimhan, Shanmugam Maheshwaran, Imad A. Abu-Yousef, Amin F. Majdalawieh, Janarthanam Rethavathi, Prince Edwin Das and Palmiro Poltronieri

Carcinogenicity (Genotox and Non Genotox) and Mutagenicity Release by Iss

Ritter Product of α-Mangostin (I A)

Q1. Normal constituent of the body: No
Q2. Contains functional groups associated with enhanced toxicity: No
Q3. Contains elements other than C,H,O,N, divalent S: No
Q5. Simply branched aliphatic hydrocarbon or a common carbohydrate: No
Q6. Benzene derivative with certain substituents: No
Q7. Heterocyclic: Yes
Q8. Lactone or cyclic diester: No
Q10. 3-membered heterocycle: No
Q11. Has a heterocyclic ring with complex substituents: Yes
Q33. Has sufficient number of sulphonate or sulphamate groups: No Class High (Class III)
Q Nongenotoxic alert? At least one alert for nongenotoxic carcinogenicity fired? No Class Negative for nongenotoxic carcinogenicity

Ritter Product of α-Mangostin (I B)

Q1. Normal constituent of the body: No
Q2. Contains functional groups associated with enhanced toxicity: Yes Class High (Class III)
Q Nongenotoxic alert? At least one alert for nongenotoxic carcinogenicity fired? No Class Negative for nongenotoxic carcinogenicity

Ritter Product of Alpha Mangostin (I C)

Q1. Normal constituent of the body: No
Q2. Contains functional groups associated with enhanced toxicity: No
Q3. Contains elements other than C,H,O,N, divalent S: No
Q5. Simply branched aliphatic hydrocarbon or a common carbohydrate: No
Q6. Benzene derivative with certain substituents: No
Q7. Heterocyclic: Yes
Q8. Lactone or cyclic diester: No
Q10. 3-membered heterocycle: No
Q11. Has a heterocyclic ring with complex substituents: Yes
Q33. Has sufficient number of sulphonate or sulphamate groups: No Class High (Class III)
Q Nongenotoxic alert? At least one alert for nongenotoxic carcinogenicity fired? No Class Negative for nongenotoxic carcinogenicity
